# Supplementary figures and images for: Adiponectin in the mammalian host influences ticks’ acquisition of the Lyme disease pathogen Borrelia
Source: PLoS Biol. 2023 Oct 20;21(10):e3002331. doi: 10.1371/journal.pbio.3002331 (PMC10619873; doi:10.1371/journal.pbio.3002331)

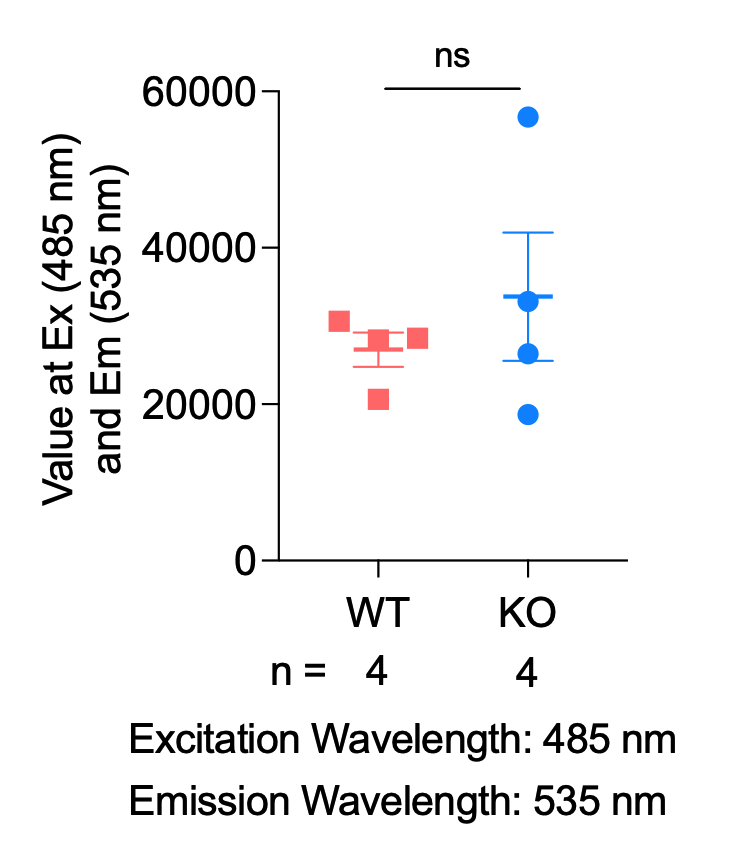

Supplement: S1 Fig — Statistical significance was assessed using a nonparametric Mann–Whitney test (ns, p > 0.05). Data underlying this figure can be found in S1 Data. (TIFF) [file pbio.3002331.s001.tiff]

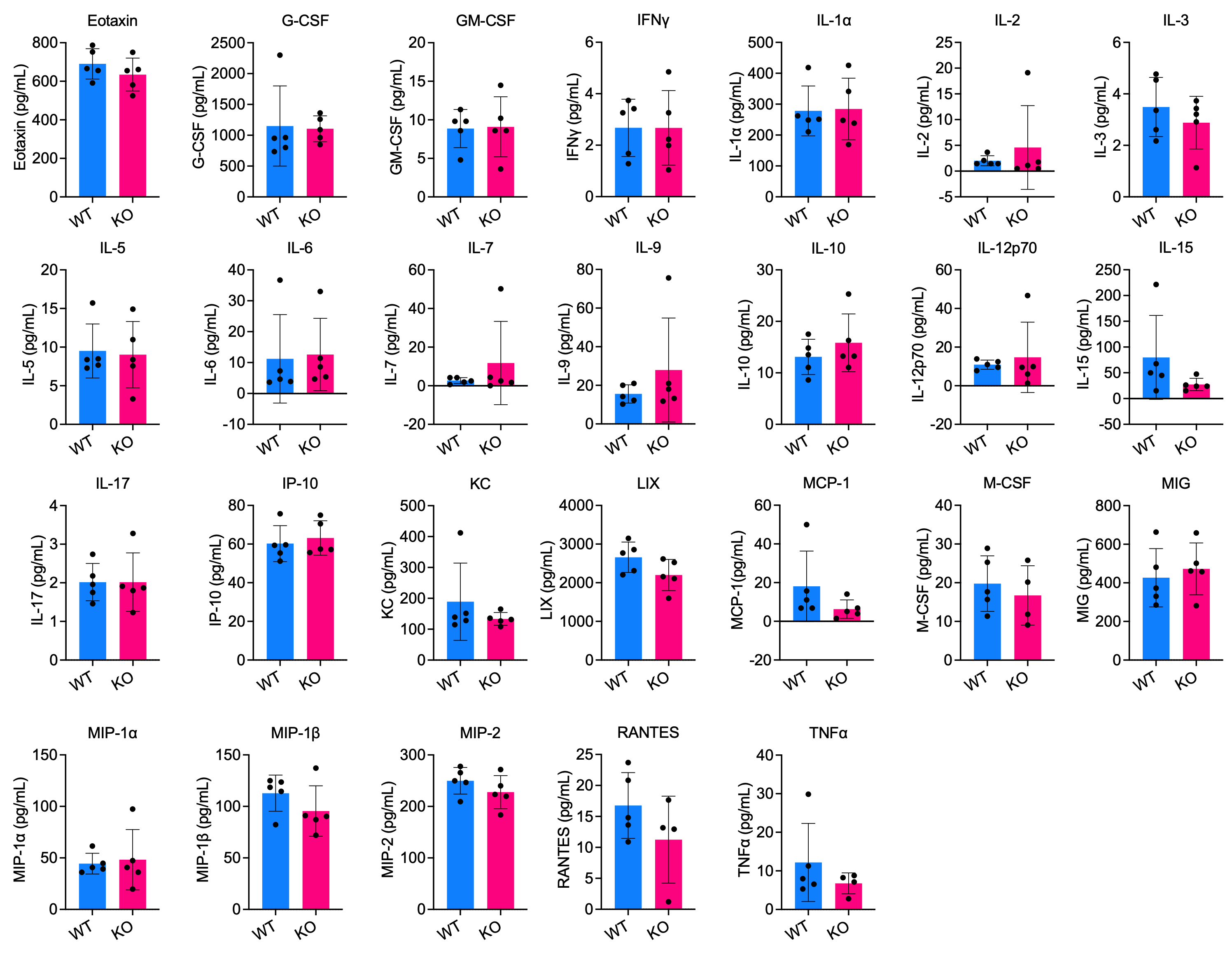

Supplement: S2 Fig — Data are represented as mean ± SD. Data underlying this figure can be found in S1 Data. (TIFF) [file pbio.3002331.s002.tiff]

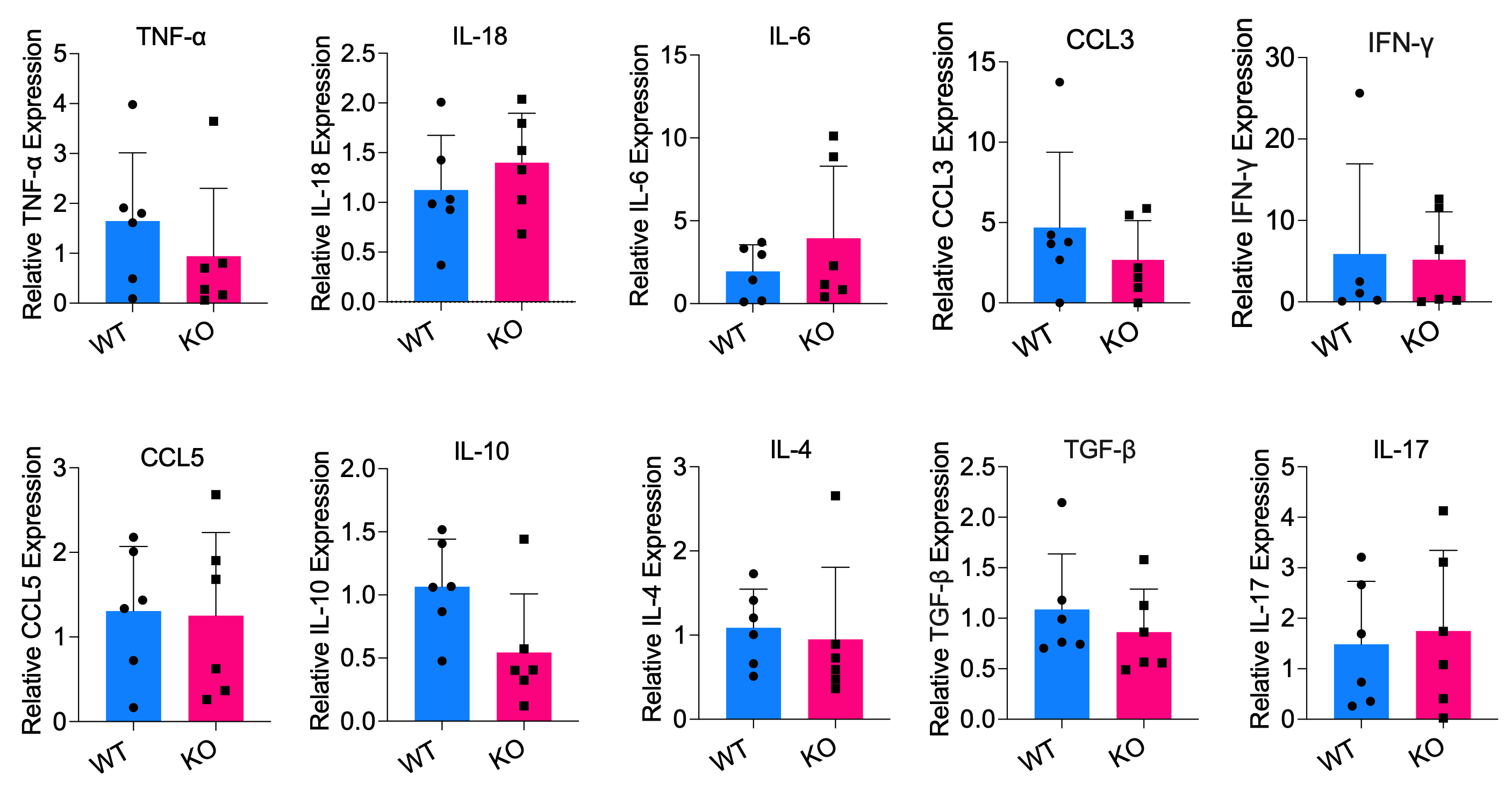

Supplement: S3 Fig — Data are represented as mean ± SD. Data underlying this figure can be found in S1 Data. (TIFF) [file pbio.3002331.s003.tiff]

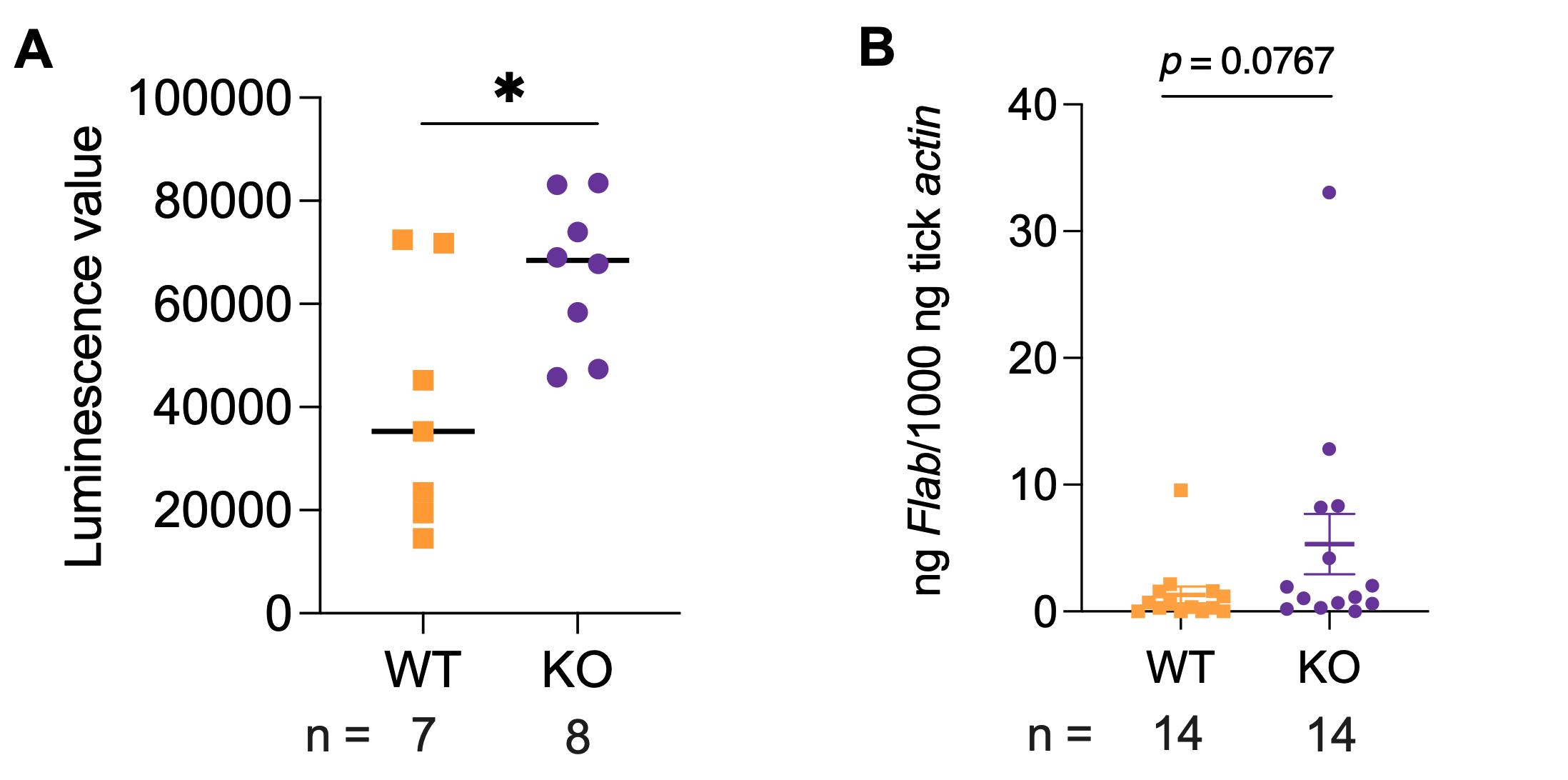

Supplement: S4 Fig — (A) B. burgdorferi in the tick bite biopsy from KO mice has higher viability than in WT mice as determined by BacTiter-Glo assay. (B) No significant difference of B. burgdorferi burden in ticks feeding on WT and KO mice after stimulating histamine release in KO mice. For all the data, statistical significance was assessed using a nonparametric Mann–Whitney test (*p < 0.05). Data underlying this figure can be found in S1 Data. (TIFF) [file pbio.3002331.s004.tiff]
