# Supplementary material for: Adiponectin in the mammalian host influences ticks’ acquisition of the Lyme disease pathogen Borrelia
Source: PLoS Biol. 2023 Oct 20;21(10):e3002331. doi: 10.1371/journal.pbio.3002331 (PMC10619873; doi:10.1371/journal.pbio.3002331)
Supplement: S1 Table — (DOCX) [file pbio.3002331.s005.docx]

**Table S1.** The differently expressed genes of transcriptome of ticks feeding on adiponectin WT and KO mice.

| **Gene** | **Annotation** | **Log2FoldChange** | **P-value** |
| --- | --- | --- | --- |
| ISCW019392 | Secreted histamine binding protein, putative | 9.64 | 0.0000 |
| ISCW019393 | Secreted histamine binding protein, putative (Fragment) | 8.72 | 0.0000 |
| ISCW007864 | Secreted histamine binding protein, putative (Fragment) | 8.44 | 0.0002 |
| ISCW005332 | Secreted salivary gland peptide, putative | 8.29 | 0.0004 |
| ISCW004075 | Uncharacterized protein (Fragment) | 8.08 | 0.0004 |
| ISCW014338 | Uncharacterized protein (Fragment) | 5.82 | 0.0000 |
| ISCW009141 | Protein PTHB1, putative (Fragment) | 5.79 | 0.0005 |
| ISCW016780 | Uncharacterized protein | 5.52 | 0.0000 |
| ISCW010812 | Glycine-rich protein GWK, putative (Fragment) | 5.01 | 0.0000 |
| ISCW019196 | Secreted protein, putative | 4.49 | 0.0003 |
| ISCW009862 | Uncharacterized protein (Fragment) | 4.48 | 0.0005 |
| ISCW002498 | SGRP-1, putative (Fragment) | 3.97 | 0.0001 |
| ISCW016593 | Cement protein, putative (Fragment) | 3.74 | 0.0000 |
| ISCW028280 | Metazoan signal recognition particle RNA | 3.14 | 0.0001 |
| ISCW005338 | CUB domaincontaining protein | 1.85 | 0.0005 |
| ISCW017436 | Uncharacterized protein | 1.14 | 0.0001 |
| ISCW006573 | HSP70 binding protein, putative (Fragment) | 1.00 | 0.0009 |
| ISCW010855 | Zinc finger protein, putative | -1.83 | 0.0009 |
| ISCW015153 | DUF5641 domain-containing protein | -2.14 | 0.0003 |
| ISCW021897 | Cell adhesion molecule, putative | -2.18 | 0.0000 |
| ISCW014724 | Salivary sulfotransferase, putative | -2.33 | 0.0001 |
| ISCW020015 | Uncharacterized protein | -2.41 | 0.0003 |
| ISCW024644 | Ficolin, putative (Secreted protein, putative) (Fragment) | -2.59 | 0.0001 |
| ISCW000699 | Uncharacterized protein | -2.73 | 0.0006 |
| ISCW002110 | Uncharacterized protein | -2.74 | 0.0009 |
| ISCW014600 | Arylsulfatase B precursor, putative | -2.78 | 0.0003 |
| ISCW020734 | DEP domain containing protein (Fragment) | -2.91 | 0.0009 |
| ISCW015204 | Serine proteinase inhibitor serpin-3, putative | -3.07 | 0.0001 |
| ISCW017243 | Monocarboxylate transporter, putative (Fragment) | -3.11 | 0.0004 |
| ISCW006689 | Uncharacterized protein | -3.14 | 0.0005 |
| ISCW024814 | Fibrinogen C-terminal domain-containing protein (Fragment) | -3.27 | 0.0000 |
| ISCW024019 | Secreted salivary gland peptide, putative (Fragment) | -3.35 | 0.0007 |
| ISCW023903 | Hebreain, putative | -3.38 | 0.0001 |
| ISCW009194 | Leucine-rich transmembrane protein, putative | -3.39 | 0.0003 |
| ISCW022256 | Carboxylic ester hydrolase | -3.43 | 0.0007 |
| ISCW002180 | AMP dependent CoA ligase, putative | -3.44 | 0.0002 |
| ISCW017305 | Cytochrome P450, putative | -3.45 | 0.0007 |
| ISCW012973 | MFS_1_like domain-containing protein | -3.48 | 0.0008 |
| ISCW018917 | Uncharacterized protein | -3.49 | 0.0002 |
| ISCW001475 | Cytochrome P450, putative | -3.51 | 0.0004 |
| ISCW002201 | SF3 helicase domain-containing protein | -3.52 | 0.0004 |
| ISCW001818 | Homocysteine S-methyltransferase, putative | -3.56 | 0.0003 |
| ISCW001476 | Cytochrome P450, putative | -3.58 | 0.0004 |
| ISCW001193 | Sodium/phosphate transporter, putative (Fragment) | -3.58 | 0.0006 |
| ISCW015396 | HMG-box transcription factor, putative | -3.61 | 0.0004 |
| ISCW004132 | Cytochrome P450, putative | -3.66 | 0.0005 |
| ISCW018031 | Homeobox protein, putative (Fragment) | -3.69 | 0.0006 |
| ISCW012091 | Uncharacterized protein | -3.69 | 0.0006 |
| ISCW019683 | Ornithine decarboxylase, putative | -3.75 | 0.0007 |
| ISCW024775 | Uncharacterized protein | -3.78 | 0.0000 |
| ISCW013352 | Proline-rich protein, putative (Fragment) | -3.81 | 0.0002 |
| ISCW019309 | Uncharacterized protein | -3.87 | 0.0004 |
| ISCW002548 | Oxoglutarate/malate carrier protein, putative | -3.96 | 0.0004 |
| ISCW024570 | Beta-ketoacyl-ACP reductase, putative (Fragment) | -4.00 | 0.0004 |
| ISCW007691 | Cystathionase, putative | -4.02 | 0.0005 |
| ISCW004734 | Chymotrypsin-C precursor, putative | -4.03 | 0.0003 |
| ISCW011828 | Uncharacterized protein | -4.04 | 0.0007 |
| ISCW000447 | Cystatin, putative | -4.19 | 0.0002 |
| ISCW013601 | Carbon-nitrogen hydrolase, putative | -4.21 | 0.0007 |
| ISCW023459 | Uncharacterized protein (Fragment) | -4.24 | 0.0001 |
| ISCW016890 | Uncharacterized protein | -4.30 | 0.0003 |
| ISCW003120 | Uncharacterized protein | -4.31 | 0.0005 |
| ISCW006263 | D-amino acid oxidase, putative | -4.35 | 0.0003 |
| ISCW017683 | Salivary sulfotransferase, putative | -4.39 | 0.0009 |
| ISCW014176 | Sodium/chloride dependent amino acid transporter, putative | -4.41 | 0.0007 |
| ISCW024685 | Secreted protein, putative (Fragment) | -4.53 | 0.0001 |
| ISCW010698 | Sodium/chloride dependent transporter, putative | -4.54 | 0.0001 |
| ISCW003063 | Elongation of very long chain fatty acids protein | -4.57 | 0.0000 |
| ISCW012637 | Dtdp-glucose 4-6-dehydratase, putative | -4.58 | 0.0006 |
| ISCW024198 | Uncharacterized protein (Fragment) | -4.60 | 0.0006 |
| ISCW020826 | Acetylcholinesterase, putative | -4.62 | 0.0004 |
| ISCW002500 | Sec14 cytosolic factor, putative | -4.64 | 0.0001 |
| ISCW002499 | Uncharacterized protein | -4.65 | 0.0001 |
| ISCW018324 | Cell death specification protein, putative | -4.67 | 0.0000 |
| ISCW017789 | Chitin synthase, putative | -4.71 | 0.0002 |
| ISCW005048 | Urease subunit gamma, putative | -4.72 | 0.0003 |
| ISCW011134 | Uncharacterized protein | -4.73 | 0.0007 |
| ISCW012204 | Sterol reductase, putative | -4.74 | 0.0002 |
| ISCW012481 | Gamma-butyrobetaine,2-oxoglutarate dioxygenase, putative | -4.75 | 0.0000 |
| ISCW023857 | Organic cation/carnitine transporter, putative | -4.78 | 0.0001 |
| ISCW013571 | Uncharacterized protein | -4.78 | 0.0005 |
| ISCW003636 | Uncharacterized protein | -4.81 | 0.0002 |
| ISCW002769 | Uncharacterized protein | -4.81 | 0.0007 |
| ISCW007756 | Glutathione S-transferase, putative | -4.83 | 0.0008 |
| ISCW014601 | O-methyltransferase, putative | -4.90 | 0.0008 |
| ISCW004189 | EF-hand domain-containing protein | -4.93 | 0.0004 |
| ISCW013274 | Uncharacterized protein | -4.96 | 0.0007 |
| ISCW021620 | RING-type domain-containing protein | -4.96 | 0.0001 |
| ISCW009114 | Uncharacterized protein | -5.03 | 0.0004 |
| ISCW024641 | Proteasome subunit alpha type, putative | -5.04 | 0.0002 |
| ISCW001207 | Uncharacterized protein (Fragment) | -5.05 | 0.0001 |
| ISCW012492 | Uncharacterized protein | -5.05 | 0.0003 |
| ISCW019880 | Cytochrome P450, putative | -5.06 | 0.0002 |
| ISCW001398 | Uncharacterized protein | -5.07 | 0.0008 |
| ISCW013259 | Uncharacterized protein | -5.09 | 0.0005 |
| ISCW007425 | Uncharacterized protein (Fragment) | -5.10 | 0.0001 |
| ISCW010544 | Salivary sulfotransferase, putative | -5.17 | 0.0001 |
| ISCW009672 | Immunoglobulin-binding protein, putative | -5.17 | 0.0000 |
| ISCW002386 | Calcium-dependent cysteine protease, putative | -5.21 | 0.0001 |
| ISCW007491 | Hebreain, putative | -5.22 | 0.0008 |
| ISCW006025 | Acid methyltransferase, putative | -5.24 | 0.0001 |
| ISCW007336 | Sulfotransferase, putative | -5.33 | 0.0008 |
| ISCW018213 | Phosphatidylinositol transfer protein SEC14, putative | -5.41 | 0.0000 |
| ISCW015006 | CRAL-TRIO domain-containing protein | -5.48 | 0.0002 |
| ISCW022593 | Located at OATL1, putative (Fragment) | -5.49 | 0.0007 |
| ISCW015098 | Phospholipid-hydroperoxide glutathione peroxidase, putative | -5.59 | 0.0001 |
| ISCW006304 | Acid methyltransferase, putative | -5.67 | 0.0005 |
| ISCW005805 | Glutathione S-transferase, putative | -5.72 | 0.0000 |
| ISCW006591 | Acyl-CoA synthetase, putative | -5.77 | 0.0001 |
| ISCW013276 | Salivary protein NPL-2, putative | -5.78 | 0.0003 |
| ISCW016395 | Cytochrome P450, putative | -5.81 | 0.0001 |
| ISCW011405 | Peroxinectin, putative | -5.82 | 0.0001 |
| ISCW009976 | Uncharacterized protein | -5.82 | 0.0002 |
| ISCW002502 | Phosphatidylinositol transfer protein sec14, putative | -5.89 | 0.0000 |
| ISCW003725 | Phosphatidylinositol transfer protein SEC14, putative | -5.90 | 0.0006 |
| ISCW024740 | Uncharacterized protein | -5.93 | 0.0000 |
| ISCW009631 | Uncharacterized protein | -5.94 | 0.0000 |
| ISCW016388 | Cytochrome P450, putative | -5.98 | 0.0000 |
| ISCW018215 | Phosphatidylinositol transfer protein SEC14, putative | -5.99 | 0.0001 |
| ISCW002194 | Secreted salivary protease inhibitor, putative (Fragment) | -6.00 | 0.0001 |
| ISCW005042 | Uncharacterized protein (Fragment) | -6.03 | 0.0000 |
| ISCW004019 | Microplusin preprotein, putative | -6.06 | 0.0000 |
| ISCW003731 | Slc2A8 protein, putative | -6.11 | 0.0000 |
| ISCW004864 | Uncharacterized protein (Fragment) | -6.12 | 0.0000 |
| ISCW007283 | Fatty-acid amide hydrolase 2-A, putative | -6.14 | 0.0008 |
| ISCW021785 | Uncharacterized protein | -6.21 | 0.0002 |
| ISCW011252 | Phosphatidylinositol transfer protein sec14, putative | -6.22 | 0.0000 |
| ISCW009481 | Chitin-binding type-2 domain-containing protein | -6.36 | 0.0000 |
| ISCW001832 | Short chain alcohol dehydrogenase, putative | -6.39 | 0.0000 |
| ISCW012412 | Kunitz domain protein, putative | -6.44 | 0.0004 |
| ISCW001401 | Uncharacterized protein | -6.48 | 0.0000 |
| ISCW013275 | Uncharacterized protein | -6.56 | 0.0000 |
| ISCW013283 | Salivary protein NPL-2, putative | -6.73 | 0.0003 |
| ISCW020587 | Glycine N-methyltransferase, putative | -6.78 | 0.0000 |
| ISCW016389 | Cytochrome P450, putative | -6.89 | 0.0000 |
| ISCW021781 | Immunoglobulin-binding protein, putative (Fragment) | -6.91 | 0.0008 |
| ISCW024013 | Serpin-2, putative | -6.91 | 0.0001 |
| ISCW015824 | Thrombospondin type 1 domaincontaining protein | -6.97 | 0.0007 |
| ISCW002193 | Secreted salivary protease inhibitor, putative | -6.97 | 0.0001 |
| ISCW013280 | Salivary protein NPL-2, putative | -7.05 | 0.0000 |
| ISCW010399 | Fatty acid synthase, putative | -7.10 | 0.0002 |
| ISCW012562 | Flavin-containing monooxygenase, putative | -7.13 | 0.0001 |
| ISCW019306 | Glutathione S-transferase, putative | -7.14 | 0.0001 |
| ISCW002487 | Secreted protein, putative | -7.15 | 0.0001 |
| ISCW004868 | Uncharacterized protein (Fragment) | -7.21 | 0.0004 |
| ISCW002675 | Trypsin, putative | -7.24 | 0.0005 |
| ISCW013271 | Uncharacterized protein | -7.24 | 0.0000 |
| ISCW022963 | Sodium-dependent multivitamin transporter, putative | -7.27 | 0.0005 |
| ISCW022249 | Acetylcholinesterase/butyrylcholinesterase, putative | -7.32 | 0.0007 |
| ISCW011016 | Serpin, putative | -7.33 | 0.0000 |
| ISCW018908 | Secreted protein, putative | -7.43 | 0.0001 |
| ISCW008687 | Uncharacterized protein | -7.47 | 0.0000 |
| ISCW008443 | Methyltransf_11 domain-containing protein | -7.51 | 0.0003 |
| ISCW011017 | Serpin-2 precursor, putative (Serpin-2, putative) | -7.58 | 0.0002 |
| ISCW007525 | Uncharacterized protein | -7.68 | 0.0000 |
| ISCW012256 | Secreted cysteine rich protein, putative (Fragment) | -7.78 | 0.0003 |
| ISCW001399 | Uncharacterized protein | -7.84 | 0.0001 |
| ISCW002941 | Uncharacterized protein | -7.85 | 0.0000 |
| ISCW024095 | Phosphatidylinositol transfer protein sec14, putative | -7.95 | 0.0000 |
| ISCW004591 | Short chain alcohol dehydrogenase, putative | -8.04 | 0.0000 |
| ISCW003519 | Methyltransf_11 domain-containing protein | -8.08 | 0.0004 |
| ISCW002501 | Phosphatidylinositol transfer protein SEC14, putative | -8.08 | 0.0000 |
| ISCW002192 | Uncharacterized protein | -8.24 | 0.0000 |
| ISCW014016 | Serine carboxypeptidase, putative | -8.28 | 0.0004 |
| ISCW007268 | Uncharacterized protein | -8.42 | 0.0000 |
| ISCW008664 | Uncharacterized protein | -8.45 | 0.0004 |
| ISCW024541 | Phosphorylase B kinase alpha, kpb1, putative | -8.45 | 0.0000 |
| ISCW021318 | Submaxillary gland androgen-regulated protein 3A, putative (Fragment) | -8.54 | 0.0000 |
| ISCW018397 | Carboxypeptidase | -8.56 | 0.0001 |
| ISCW008007 | Uncharacterized protein | -8.62 | 0.0000 |
| ISCW008542 | Uncharacterized protein | -8.63 | 0.0000 |
| ISCW010729 | Uncharacterized protein | -8.70 | 0.0004 |
| ISCW013356 | Uncharacterized protein | -8.81 | 0.0001 |
| ISCW002674 | Serin proteinase, putative | -8.81 | 0.0000 |
| ISCW016397 | Cytochrome P450, putative | -8.90 | 0.0000 |
| ISCW003785 | Cuticle protein, putative | -8.97 | 0.0002 |
| ISCW015350 | Peptidase, putative | -8.98 | 0.0000 |
| ISCW022672 | Cytochrome P-450, putative | -9.05 | 0.0000 |
| ISCW018906 | Secreted protein, putative | -9.15 | 0.0000 |
| ISCW013195 | Secreted protein, putative | -9.19 | 0.0000 |
| ISCW015837 | Uncharacterized protein (Fragment) | -9.21 | 0.0004 |
| ISCW018910 | Uncharacterized protein | -9.28 | 0.0000 |
| ISCW021492 | Acid methyltransferase, putative | -9.31 | 0.0002 |
| ISCW018900 | Uncharacterized protein | -9.58 | 0.0001 |
| ISCW013279 | Uncharacterized protein | -9.64 | 0.0000 |
| ISCW000508 | Secreted protein, putative | -9.67 | 0.0003 |
| ISCW018904 | Uncharacterized protein | -9.68 | 0.0005 |
| ISCW004625 | Anoctamin (Fragment) | -9.77 | 0.0000 |
| ISCW010024 | Fibronectin type III domaincontaining protein | -9.84 | 0.0000 |
| ISCW019391 | Uncharacterized protein | -9.88 | 0.0000 |
| ISCW011411 | Monooxygenase, putative (Fragment) | -9.93 | 0.0000 |
| ISCW002037 | Cystatin, putative | -9.97 | 0.0000 |
| ISCW001469 | Secreted salivary gland peptide, putative | -9.99 | 0.0002 |
| ISCW011357 | Cytochrome P450, putative | -10.03 | 0.0000 |
| ISCW014533 | Uncharacterized protein (Fragment) | -10.04 | 0.0001 |
| ISCW023169 | Secreted salivary gland peptide, putative (Fragment) | -10.13 | 0.0004 |
| ISCW001782 | Uncharacterized protein | -10.21 | 0.0008 |
| ISCW009572 | Uncharacterized protein (Fragment) | -10.26 | 0.0000 |
| ISCW009462 | Short chain type dehydrogenase, putative | -10.41 | 0.0000 |
| ISCW011970 | Phosphatidylinositol transfer protein sec14, putative | -10.43 | 0.0000 |
| ISCW000188 | Uncharacterized protein | -10.43 | 0.0000 |
| ISCW002216 | Cystatin, putative | -11.40 | 0.0002 |
| ISCW002191 | Uncharacterized protein | -12.65 | 0.0000 |
| ISCW023575 | Uncharacterized protein | -12.72 | 0.0000 |
| ISCW004553 | Cuticle protein, putative | -12.80 | 0.0000 |
| ISCW013351 | Uncharacterized protein | -13.15 | 0.0000 |
| ISCW007380 | Cytochrome p450, putative | -14.08 | 0.0000 |
| ISCW009079 | Uncharacterized protein | -24.73 | 0.0000 |
| ISCW021419 | Uncharacterized protein (Fragment) | -25.28 | 0.0000 |
| ISCW022944 | Secreted protein, putative | -25.75 | 0.0000 |
| ISCW007369 | Acid methyltransferase, putative | -25.87 | 0.0000 |
| ISCW005191 | Secreted glycine rich protein, putative (Fragment) | -26.30 | 0.0000 |
| ISCW009487 | Uncharacterized protein | -26.81 | 0.0000 |
| ISCW001393 | Uncharacterized protein | -27.39 | 0.0000 |
| ISCW014017 | Uncharacterized protein | -27.49 | 0.0000 |
| ISCW003712 | Paramyosin, putative (Fragment) | -28.10 | 0.0000 |
| ISCW001781 | Cuticle protein, putative | -28.46 | 0.0000 |
| ISCW008564 | Cuticle protein, putative | -28.81 | 0.0000 |
| ISCW008565 | Cuticle protein, putative | -29.20 | 0.0000 |
| ISCW003950 | Chitinase, putative | -29.43 | 0.0000 |
| ISCW007054 | Uncharacterized protein (Fragment) | -30.82 | 0.0000 |

“-” indicates downregulation of genes in the ticks feeding on adiponectin WT mice compared to that feeding on KO mice.
