# Supplementary material for: Adiponectin in the mammalian host influences ticks’ acquisition of the Lyme disease pathogen Borrelia
Source: PLoS Biol. 2023 Oct 20;21(10):e3002331. doi: 10.1371/journal.pbio.3002331 (PMC10619873; doi:10.1371/journal.pbio.3002331)
Supplement: S2 Table — (DOCX) [file pbio.3002331.s006.docx]

**Table S2.** The differently expressed genes of transcriptome of WT and KO murine skin during tick bite.

| **Gene symbol** | **Gene name** | **Fold change** | **P-value** | |
| --- | --- | --- | --- | --- |
| Mup22 | major urinary protein 22 (Mup22) | -9.03 | | 0.0494 |
| Lars2 | leucyl-tRNA synthetase, mitochondrial (Lars2) | -8.85 | | 0.0081 |
| Areg | Amphiregulin (Areg) | -6.15 | | 0.0106 |
| H2-Q10 | histocompatibility 2, Q region locus 10 (H2-Q10) | -5.51 | | 0.0008 |
| Fam83a | family with sequence similarity 83, member A (Fam83a) | -3.85 | | 0.0134 |
| Thrsp | thyroid hormone responsive (Thrsp) | -3.81 | | 0.0101 |
| Gm9780 | predicted gene 9780 (Gm9780) | -3.81 | | 0.0003 |
| Plac9b | N/A | -3.81 | | 0.0003 |
| Prrt2 | proline-rich transmembrane protein 2 (Prrt2) | -3.59 | | 0.0124 |
| Mmp13 | matrix metallopeptidase 13 (Mmp13) | -3.42 | | 0.0494 |
| Unc5a | unc-5 netrin receptor A (Unc5a) | -3.36 | | 0.0111 |
| Chn1 | chimerin 1 (Chn1) | -3.25 | | 0.0311 |
| Pthlh | parathyroid hormone-like peptide (Pthlh) | -3.24 | | 0.0293 |
| Lilra6 | leukocyte immunoglobulin-like receptor, subfamily A (with TM domain), member 6 (Lilra6) | -3.22 | | 0.0305 |
| Nell2 | NEL-like 2 (Nell2) | -3.08 | | 0.0243 |
| Tmem254b | transmembrane protein 268 (Tmem268) | -3.02 | | 0.0001 |
| Aldh1a7 | aldehyde dehydrogenase family 1, subfamily A7 (Aldh1a7) | -2.81 | | 0.0190 |
| Fos | FBJ osteosarcoma oncogene (Fos) | -2.76 | | 0.0292 |
| Smarcd3 | SWI/SNF related, matrix associated, actin dependent regulator of chromatin, subfamily d, member 3 (Smarcd3) | -2.71 | | 0.0011 |
| Cdo1 | cysteine dioxygenase 1, cytosolic (Cdo1) | -2.63 | | 0.0100 |
| Osr2 | odd-skipped related 2 (Osr2) | -2.62 | | 0.0159 |
| Gjb2 | gap junction protein, beta 2 (Gjb2) | -2.55 | | 0.0007 |
| Myh8 | myosin, heavy polypeptide 8, skeletal muscle, perinatal (Myh8) | -2.54 | | 0.0109 |
| Mrap | melanocortin 2 receptor accessory protein (Mrap) | -2.53 | | 0.0406 |
| Bdkrb1 | bradykinin receptor, beta 1 (Bdkrb1) | -2.51 | | 0.0263 |
| Serpine1 | serine (or cysteine) peptidase inhibitor, clade E, member 1 (Serpine1) | -2.48 | | 0.0107 |
| Gm20544 | predicted gene 20544 (Gm20544) | -2.44 | | 0.0249 |
| Tmem254a | transmembrane protein 267 (Tmem267) | -2.42 | | 0.0037 |
| Mtfp1 | mitochondrial fission process 1 (Mtfp1) | -2.38 | | 0.0109 |
| Ugt1a6b | UDP glucuronosyltransferase 1 family, polypeptide A6B (Ugt1a6b) | -2.36 | | 0.0400 |
| Retn | resistin (Retn) | -2.34 | | 0.0151 |
| Cmpk2 | cytidine monophosphate (UMP-CMP) kinase 2, mitochondrial (Cmpk2) | -2.33 | | 0.0387 |
| Dyrk3 | dual-specificity tyrosine- (Y)-phosphorylation regulated kinase 3 (Dyrk3) | -2.33 | | 0.0372 |
| 1700037C18Rik | RIKEN cDNA 1110028F11 gene (1110028F11Rik) | -2.32 | | 0.0310 |
| Aamdc | adipogenesis associated Mth938 domain containing (Aamdc) | -2.32 | | 0.0041 |
| Spp1 | secreted phosphoprotein 1 (Spp1) | -2.32 | | 0.0302 |
| P2rx2 | purinergic receptor P2X, ligand-gated ion channel, 2 (P2rx2) | -2.31 | | 0.0329 |
| Rida | reactive intermediate imine deaminase A homolog (Rida) | -2.31 | | 0.0075 |
| Tbx3 | T-box 3 (Tbx3) | -2.26 | | 0.0385 |
| Dusp26 | dual specificity phosphatase 26 (putative) (Dusp26) | -2.23 | | 0.0378 |
| Pnpla3 | patatin-like phospholipase domain containing 3 (Pnpla3) | -2.22 | | 0.0051 |
| Lrrn3 | leucine rich repeat protein 3, neuronal (Lrrn3) | -2.21 | | 0.0497 |
| Tmem45b | transmembrane protein 45b (Tmem45b) | -2.20 | | 0.0215 |
| Tmem179 | transmembrane protein 179 (Tmem179) | -2.18 | | 0.0430 |
| Gpd1 | glycerol-3-phosphate dehydrogenase 1 (soluble) (Gpd1) | -2.18 | | 0.0103 |
| Adgrg3 | adhesion G protein-coupled receptor G3 (Adgrg3) | -2.14 | | 0.0381 |
| Pkhd1l1 | polycystic kidney and hepatic disease 1-like 1 (Pkhd1l1) | -2.12 | | 0.0175 |
| Kbtbd12 | kelch repeat and BTB (POZ) domain containing 12 (Kbtbd12) | -2.12 | | 0.0470 |
| Lrrc14b | leucine rich repeat containing 14B (Lrrc14b) | -2.12 | | 0.0428 |
| Myct1 | myc target 1 (Myct1) | -2.12 | | 0.0359 |
| Hspa1l | heat shock protein 1-like (Hspa1l) | -2.12 | | 0.0055 |
| Pctp | phosphatidylcholine transfer protein (Pctp) | -2.12 | | 0.0012 |
| Serpina3j | serine (or cysteine) peptidase inhibitor, clade A (alpha-1 antiproteinase, antitrypsin), member 3J (Serpina3j) | -2.11 | | 0.0172 |
| Ppp1r1a | protein phosphatase 1, regulatory inhibitor subunit 1A (Ppp1r1a) | -2.10 | | 0.0139 |
| Trarg1 | trafficking regulator of GLUT4 (SLC2A4) 1 (Trarg1) | -2.05 | | 0.0265 |
| Ptgfr | prostaglandin F receptor (Ptgfr) | -2.05 | | 0.0102 |
| Acvr1c | activin A receptor, type IC (Acvr1c) | -2.05 | | 0.0275 |
| Hacd1 | 3-hydroxyacyl-CoA dehydratase 1 (Hacd1) | -2.05 | | 0.0354 |
| Gm6710 | predicted gene 6710 (Gm6710) | -2.04 | | 0.0134 |
| Inhba | inhibin beta-A (Inhba) | -2.03 | | 0.0322 |
| Tmem254c | transmembrane protein 38B (Tmem38b) | -2.02 | | 0.0104 |
| Serpina3c | serine (or cysteine) peptidase inhibitor, clade A, member 3C (Serpina3c) | -2.00 | | 0.0335 |
| Rai2 | retinoic acid induced 2 (Rai2) | -1.99 | | 0.0181 |
| Plet1 | placenta expressed transcript 1 (Plet1) | -1.98 | | 0.0291 |
| Slc22a3 | solute carrier family 22 (organic cation transporter), member 3 (Slc22a3) | -1.97 | | 0.0385 |
| Tnfrsf11b | tumor necrosis factor receptor superfamily, member 11b (osteoprotegerin) (Tnfrsf11b) | -1.97 | | 0.0057 |
| Gsta3 | glutathione S-transferase, alpha 3 (Gsta3) | -1.95 | | 0.0393 |
| Dusp1 | dual specificity phosphatase 1 (Dusp1) | -1.92 | | 0.0396 |
| Camk2n1 | calcium/calmodulin-dependent protein kinase II inhibitor 1 (Camk2n1) | -1.90 | | 0.0044 |
| Nnmt | nicotinamide N-methyltransferase (Nnmt) | -1.90 | | 0.0264 |
| B4galnt2 | beta-1,4-N-acetyl-galactosaminyl transferase 2 (B4galnt2) | -1.90 | | 0.0082 |
| Ddit3 | DNA-damage inducible transcript 3 (Ddit3) | -1.88 | | 0.0127 |
| Cxcr4 | chemokine (C-X-C motif) receptor 4 (Cxcr4) | -1.88 | | 0.0167 |
| Procr | protein C receptor, endothelial (Procr) | -1.88 | | 0.0339 |
| Lyz1 | lysozyme 1 (Lyz1) | -1.85 | | 0.0074 |
| Slc2a3 | solute carrier family 2 (facilitated glucose transporter), member 3 (Slc2a3) | -1.84 | | 0.0450 |
| Pacrgl | PARK2 co-regulated-like (Pacrgl) | -1.84 | | 0.0363 |
| Klhdc1 | kelch domain containing 1 (Klhdc1) | -1.84 | | 0.0172 |
| Galnt15 | polypeptide N-acetylgalactosaminyltransferase 15 (Galnt15) | -1.83 | | 0.0161 |
| Gem | GTP binding protein (gene overexpressed in skeletal muscle) (Gem) | -1.83 | | 0.0186 |
| Robo4 | roundabout guidance receptor 4 (Robo4) | -1.82 | | 0.0331 |
| Gimap8 | GTPase, IMAP family member 8 (Gimap8) | -1.82 | | 0.0495 |
| Myoz2 | myozenin 2 (Myoz2) | -1.80 | | 0.0047 |
| Islr2 | immunoglobulin superfamily containing leucine-rich repeat 2 (Islr2) | -1.79 | | 0.0475 |
| Fabp4 | fatty acid binding protein 4, adipocyte (Fabp4) | -1.79 | | 0.0255 |
| H2-T24 | histocompatibility 2, T region locus 24 (H2-T24) | -1.76 | | 0.0476 |
| Grb14 | growth factor receptor bound protein 14 (Grb14) | -1.76 | | 0.0304 |
| Hoxa3 | homeobox A3 (Hoxa3) | -1.75 | | 0.0362 |
| Tspan12 | tetraspanin 12 (Tspan12) | -1.75 | | 0.0226 |
| Jsrp1 | junctional sarcoplasmic reticulum protein 1 (Jsrp1) | -1.75 | | 0.0145 |
| Krt17 | keratin 17 (Krt17) | -1.75 | | 0.0001 |
| Scml2 | Scm polycomb group protein like 2 (Scml2) | -1.74 | | 0.0153 |
| Lpl | lipoprotein lipase (Lpl) | -1.74 | | 0.0192 |
| Jrk | jerky (Jrk) | -1.74 | | 0.0054 |
| Fam167a | family with sequence similarity 167, member A (Fam167a) | -1.73 | | 0.0420 |
| Pparg | peroxisome proliferator activated receptor gamma (Pparg) | -1.73 | | 0.0160 |
| Angptl7 | angiopoietin-like 7 (Angptl7) | -1.73 | | 0.0065 |
| Anapc10 | anaphase promoting complex subunit 10 (Anapc10) | -1.73 | | 0.0309 |
| Chpt1 | choline phosphotransferase 1 (Chpt1) | -1.71 | | 0.0045 |
| Aoc3 | amine oxidase, copper containing 3 (Aoc3) | -1.71 | | 0.0374 |
| Agpat2 | 1-acylglycerol-3-phosphate O-acyltransferase 2 (lysophosphatidic acid acyltransferase, beta) (Agpat2) | -1.68 | | 0.0070 |
| Dusp6 | dual specificity phosphatase 6 (Dusp6) | -1.68 | | 0.0191 |
| Nr4a1 | nuclear receptor subfamily 4, group A, member 1 (Nr4a1) | -1.67 | | 0.0019 |
| Fosb | FBJ osteosarcoma oncogene B (Fosb) | -1.67 | | 0.0338 |
| Zfp867 | zinc finger protein 867 (Zfp867) | -1.66 | | 0.0190 |
| Dpep1 | dipeptidase 1 (Dpep1) | -1.66 | | 0.0313 |
| Egr1 | early growth response 1 (Egr1) | -1.66 | | 0.0106 |
| Apod | apolipoprotein D (Apod) | -1.64 | | 0.0023 |
| Hsd11b1 | hydroxysteroid 11-beta dehydrogenase 1 (Hsd11b1) | -1.64 | | 0.0214 |
| Klf2 | Kruppel-like factor 2 (lung) (Klf2) | -1.64 | | 0.0081 |
| 4931406C07Rik | RIKEN cDNA 4930412O13 gene (4930412O13Rik) | -1.63 | | 0.0383 |
| Mme | membrane metallo endopeptidase (Mme) | -1.63 | | 0.0127 |
| Slc16a3 | solute carrier family 16 (monocarboxylic acid transporters), member 3 (Slc16a3) | -1.62 | | 0.0067 |
| Pxdc1 | PX domain containing 1 (Pxdc1) | -1.62 | | 0.0168 |
| Nnt | nicotinamide nucleotide transhydrogenase (Nnt) | -1.61 | | 0.0094 |
| Tek | TEK receptor tyrosine kinase (Tek) | -1.61 | | 0.0087 |
| Nectin3 | nectin cell adhesion molecule 3 (Nectin3) | -1.60 | | 0.0309 |
| Zfp874b | zinc finger protein 874b (Zfp874b) | -1.60 | | 0.0127 |
| Myl1 | myosin, light polypeptide 1 (Myl1) | -1.60 | | 0.0142 |
| Atg4c | autophagy related 4C, cysteine peptidase (Atg4c) | -1.60 | | 0.0156 |
| Pcx | pyruvate carboxylase (Pcx) | -1.59 | | 0.0012 |
| Cyp2f2 | cytochrome P450, family 2, subfamily f, polypeptide 2 (Cyp2f2) | -1.59 | | 0.0113 |
| Snx16 | sorting nexin 16 (Snx16) | -1.59 | | 0.0188 |
| Musk | muscle, skeletal, receptor tyrosine kinase (Musk) | -1.58 | | 0.0468 |
| Homer3 | homer scaffolding protein 3 (Homer3) | -1.58 | | 0.0278 |
| Car3 | carbonic anhydrase 3 (Car3) | -1.57 | | 0.0287 |
| Phka1 | phosphorylase kinase alpha 1 (Phka1) | -1.57 | | 0.0123 |
| Rgs2 | regulator of G-protein signaling 2 (Rgs2) | -1.53 | | 0.0083 |
| Sdc2 | syndecan 2 (Sdc2) | -1.53 | | 0.0051 |
| Cavin2 | caveolae associated 2 (Cavin2) | -1.53 | | 0.0483 |
| Gjb6 | gap junction protein, beta 6 (Gjb6) | -1.52 | | 0.0451 |
| Utp23 | UTP23 small subunit processome component (Utp23) | -1.52 | | 0.0227 |
| Mrps6 | mitochondrial ribosomal protein S6 (Mrps6) | -1.52 | | 0.0425 |
| Igf2 | insulin-like growth factor 2 (Igf2) | -1.51 | | 0.0145 |
| Tmem268 | transmembrane protein 268 (Tmem268) | -1.51 | | 0.0373 |
| Entpd4b | ectonucleoside triphosphate diphosphohydrolase 4B (Entpd4b) | -1.51 | | 0.0255 |
| Pmepa1 | prostate transmembrane protein, androgen induced 1 (Pmepa1) | -1.50 | | 0.0228 |
| Zbtb16 | zinc finger and BTB domain containing 16 (Zbtb16) | -1.50 | | 0.0239 |
| Rhobtb3 | Rho-related BTB domain containing 3 (Rhobtb3) | -1.49 | | 0.0260 |
| Tnfaip3 | tumor necrosis factor, alpha-induced protein 3 (Tnfaip3) | -1.49 | | 0.0315 |
| Tshz3 | teashirt zinc finger family member 3 (Tshz3) | -1.49 | | 0.0246 |
| Plk2 | polo like kinase 2 (Plk2) | -1.48 | | 0.0486 |
| Klhl2 | kelch-like 2, Mayven (Klhl2) | -1.48 | | 0.0138 |
| Prg4 | proteoglycan 4 (megakaryocyte stimulating factor, articular superficial zone protein) (Prg4) | -1.48 | | 0.0500 |
| Tcap | titin-cap (Tcap) | -1.48 | | 0.0315 |
| Tmem38b | transmembrane protein 38B (Tmem38b) | -1.47 | | 0.0104 |
| Casq1 | calsequestrin 1 (Casq1) | -1.47 | | 0.0335 |
| Fasn | fatty acid synthase (Fasn) | -1.47 | | 0.0005 |
| Lrp12 | low density lipoprotein-related protein 12 (Lrp12) | -1.46 | | 0.0147 |
| Nfkbia | nuclear factor of kappa light polypeptide gene enhancer in B cells inhibitor, alpha (Nfkbia) | -1.46 | | 0.0387 |
| Lins1 | lines homolog 1 (Lins1) | -1.46 | | 0.0480 |
| Rfx3 | regulatory factor X, 3 (influences HLA class II expression) (Rfx3) | -1.46 | | 0.0485 |
| Slit3 | slit guidance ligand 3 (Slit3) | -1.46 | | 0.0288 |
| Fxyd1 | FXYD domain-containing ion transport regulator 1 (Fxyd1) | -1.46 | | 0.0369 |
| Stxbp6 | syntaxin binding protein 6 (amisyn) (Stxbp6) | -1.45 | | 0.0418 |
| Lyve1 | lymphatic vessel endothelial hyaluronan receptor 1 (Lyve1) | -1.45 | | 0.0217 |
| Rhob | ras homolog family member B (Rhob) | -1.45 | | 0.0260 |
| Ntmt1 | N-terminal Xaa-Pro-Lys N-methyltransferase 1 (Ntmt1) | -1.45 | | 0.0303 |
| Mmrn1 | multimerin 1 (Mmrn1) | -1.45 | | 0.0448 |
| Snta1 | syntrophin, acidic 1 (Snta1) | -1.45 | | 0.0284 |
| Eif4ebp1 | eukaryotic translation initiation factor 4E binding protein 1 (Eif4ebp1) | -1.45 | | 0.0264 |
| Mrpl39 | mitochondrial ribosomal protein L39 (Mrpl39) | -1.45 | | 0.0313 |
| Zdhhc13 | zinc finger, DHHC domain containing 13 (Zdhhc13) | -1.45 | | 0.0378 |
| Itga2 | integrin alpha 2 (Itga2) | -1.44 | | 0.0125 |
| Fkbp5 | FK506 binding protein 5 (Fkbp5) | -1.43 | | 0.0348 |
| Ccdc22 | coiled-coil domain containing 22 (Ccdc22) | -1.43 | | 0.0277 |
| Gm7429 | predicted pseudogene 7429 (Gm7429) | -1.43 | | 0.0147 |
| Mosmo | modulator of smoothened (Mosmo) | -1.43 | | 0.0071 |
| Cd36 | CD36 molecule (Cd36) | -1.43 | | 0.0361 |
| Cldn23 | claudin 23 (Cldn23) | -1.43 | | 0.0112 |
| Atf3 | activating transcription factor 3 (Atf3) | -1.43 | | 0.0368 |
| Scd1 | stearoyl-Coenzyme A desaturase 1 (Scd1) | -1.42 | | 0.0295 |
| Cygb | cytoglobin (Cygb) | -1.42 | | 0.0379 |
| Klf9 | Kruppel-like factor 9 (Klf9) | -1.41 | | 0.0337 |
| Myh1 | myosin, heavy polypeptide 1, skeletal muscle, adult (Myh1) | -1.41 | | 0.0091 |
| Smpx | small muscle protein, X-linked (Smpx) | -1.41 | | 0.0296 |
| Anapc13 | anaphase promoting complex subunit 13 (Anapc13) | -1.41 | | 0.0437 |
| Mylpf | myosin light chain, phosphorylatable, fast skeletal muscle (Mylpf) | -1.41 | | 0.0404 |
| Pde3b | phosphodiesterase 3B, cGMP-inhibited (Pde3b) | -1.41 | | 0.0470 |
| Bmp3 | bone morphogenetic protein 3 (Bmp3) | -1.40 | | 0.0325 |
| Ednra | endothelin receptor type A (Ednra) | -1.40 | | 0.0495 |
| Man1a | mannosidase 1, alpha (Man1a) | -1.39 | | 0.0334 |
| Hs3st3b1 | heparan sulfate (glucosamine) 3-O-sulfotransferase 3B1 (Hs3st3b1) | -1.39 | | 0.0222 |
| Rilpl1 | Rab interacting lysosomal protein-like 1 (Rilpl1) | -1.39 | | 0.0333 |
| Irs2 | insulin receptor substrate 2 (Irs2) | -1.39 | | 0.0123 |
| Gfpt2 | glutamine fructose-6-phosphate transaminase 2 (Gfpt2) | -1.38 | | 0.0409 |
| Creld2 | cysteine-rich with EGF-like domains 2 (Creld2) | -1.38 | | 0.0448 |
| Acss2 | acyl-CoA synthetase short-chain family member 2 (Acss2) | -1.38 | | 0.0476 |
| Cacna1s | calcium channel, voltage-dependent, L type, alpha 1S subunit (Cacna1s) | -1.38 | | 0.0267 |
| Prelp | proline arginine-rich end leucine-rich repeat (Prelp) | -1.37 | | 0.0456 |
| Ackr3 | atypical chemokine receptor 3 (Ackr3) | -1.37 | | 0.0429 |
| Dnajb9 | DnaJ heat shock protein family (Hsp40) member B9 (Dnajb9) | -1.37 | | 0.0461 |
| Etfrf1 | electron transfer flavoprotein regulatory factor 1 (Etfrf1) | -1.37 | | 0.0442 |
| Slc15a4 | solute carrier family 15, member 4 (Slc15a4) | -1.36 | | 0.0313 |
| Tshz2 | teashirt zinc finger family member 2 (Tshz2) | -1.36 | | 0.0135 |
| Mb | myoglobin (Mb) | -1.36 | | 0.0247 |
| Cpeb3 | cytoplasmic polyadenylation element binding protein 3 (Cpeb3) | -1.36 | | 0.0259 |
| Asf1a | anti-silencing function 1A histone chaperone (Asf1a) | -1.35 | | 0.0369 |
| Ptprb | protein tyrosine phosphatase, receptor type, B (Ptprb) | -1.35 | | 0.0314 |
| Suco | SUN domain containing ossification factor (Suco) | -1.35 | | 0.0227 |
| Mpc1 | mitochondrial pyruvate carrier 1 (Mpc1) | -1.35 | | 0.0470 |
| Klf6 | Kruppel-like factor 6 (Klf6) | -1.35 | | 0.0102 |
| Amot | angiomotin (Amot) | -1.34 | | 0.0375 |
| Tmed4 | transmembrane p24 trafficking protein 4 (Tmed4) | -1.34 | | 0.0211 |
| Fbxo30 | F-box protein 30 (Fbxo30) | -1.34 | | 0.0186 |
| Kif21a | kinesin family member 21A (Kif21a) | -1.34 | | 0.0234 |
| Stk17b | serine/threonine kinase 17b (apoptosis-inducing) (Stk17b) | -1.34 | | 0.0118 |
| Lratd2 | LRAT domain containing 1 (Lratd2) | -1.33 | | 0.0364 |
| Hmga1 | high mobility group AT-hook 1 (Hmga1) | -1.32 | | 0.0192 |
| Ggps1 | geranylgeranyl diphosphate synthase 1 (Ggps1) | -1.32 | | 0.0216 |
| Ppat | phosphoribosyl pyrophosphate amidotransferase (Ppat) | -1.32 | | 0.0463 |
| Echdc1 | enoyl Coenzyme A hydratase domain containing 1 (Echdc1) | -1.32 | | 0.0129 |
| Lamb3 | laminin, beta 3 (Lamb3) | -1.31 | | 0.0156 |
| Atp13a3 | ATPase type 13A3 (Atp13a3) | -1.31 | | 0.0166 |
| St3gal1 | ST3 beta-galactoside alpha-2,3-sialyltransferase 1 (St3gal1) | -1.31 | | 0.0489 |
| Lysmd3 | LysM, putative peptidoglycan-binding, domain containing 3 (Lysmd3) | -1.31 | | 0.0440 |
| Pitpnc1 | phosphatidylinositol transfer protein, cytoplasmic 1 (Pitpnc1) | -1.31 | | 0.0356 |
| Yae1d1 | Yae1 domain containing 1 (Yae1d1) | -1.31 | | 0.0420 |
| Ckmt2 | creatine kinase, mitochondrial 2 (Ckmt2) | -1.31 | | 0.0417 |
| Per1 | period circadian clock 1 (Per1) | -1.30 | | 0.0206 |
| Prdx3 | peroxiredoxin 3 (Prdx3) | -1.30 | | 0.0314 |
| Numb | NUMB endocytic adaptor protein (Numb) | -1.30 | | 0.0238 |
| Parvb | parvin, beta (Parvb) | -1.30 | | 0.0404 |
| Pou6f1 | POU domain, class 6, transcription factor 1 (Pou6f1) | -1.30 | | 0.0475 |
| Sspn | sarcospan (Sspn) | -1.30 | | 0.0434 |
| Gm13363 | predicted gene 13363 (Gm13363) | -1.30 | | 0.0272 |
| Myadm | myeloid-associated differentiation marker (Myadm) | -1.29 | | 0.0114 |
| Mier3 | MIER family member 3 (Mier3) | -1.29 | | 0.0398 |
| Atp1a2 | ATPase, Na+/K+ transporting, alpha 2 polypeptide (Atp1a2) | -1.29 | | 0.0422 |
| Ppp1r2 | protein phosphatase 1, regulatory inhibitor subunit 2 (Ppp1r2) | -1.29 | | 0.0104 |
| R3hdm1 | R3H domain containing 1 (R3hdm1) | -1.29 | | 0.0215 |
| Pithd1 | PITH (C-terminal proteasome-interacting domain of thioredoxin-like) domain containing 1 (Pithd1) | -1.28 | | 0.0396 |
| Nrip1 | nuclear receptor interacting protein 1 (Nrip1) | -1.28 | | 0.0184 |
| Ppfibp1 | PTPRF interacting protein, binding protein 1 (liprin beta 1) (Ppfibp1) | -1.27 | | 0.0156 |
| Rap1a | RAS-related protein 1a (Rap1a) | -1.27 | | 0.0328 |
| Proser1 | proline and serine rich 1 (Proser1) | -1.27 | | 0.0395 |
| Zfp655 | zinc finger protein 655 (Zfp655) | -1.27 | | 0.0358 |
| Fam129a | family with sequence similarity 129, member A (Fam129a) | -1.27 | | 0.0467 |
| Cdk7 | cyclin-dependent kinase 7 (Cdk7) | -1.27 | | 0.0303 |
| Camsap2 | calmodulin regulated spectrin-associated protein family, member 2 (Camsap2) | -1.26 | | 0.0277 |
| Anapc4 | anaphase promoting complex subunit 4 (Anapc4) | -1.26 | | 0.0452 |
| Rassf3 | Ras association (RalGDS/AF-6) domain family member 3 (Rassf3) | -1.26 | | 0.0296 |
| Mcl1 | myeloid cell leukemia sequence 1 (Mcl1) | -1.26 | | 0.0198 |
| Dusp3 | dual specificity phosphatase 3 (vaccinia virus phosphatase VH1-related) (Dusp3) | -1.26 | | 0.0351 |
| Zdhhc21 | zinc finger, DHHC domain containing 21 (Zdhhc21) | -1.26 | | 0.0204 |
| Sec23b | SEC23 homolog B, COPII coat complex component (Sec23b) | -1.25 | | 0.0485 |
| Mafk | v-maf musculoaponeurotic fibrosarcoma oncogene family, protein K (avian) (Mafk) | -1.25 | | 0.0482 |
| Ccdc50 | coiled-coil domain containing 50 (Ccdc50) | -1.25 | | 0.0332 |
| Ube2v2 | ubiquitin-conjugating enzyme E2 variant 2 (Ube2v2) | -1.24 | | 0.0481 |
| Arl13b | ADP-ribosylation factor-like 13B (Arl13b) | -1.24 | | 0.0448 |
| Lrrfip1 | leucine rich repeat (in FLII) interacting protein 1 (Lrrfip1) | -1.24 | | 0.0214 |
| Kdm7a | lysine (K)-specific demethylase 7A (Kdm7a) | -1.24 | | 0.0102 |
| Utp3 | UTP3 small subunit processome component (Utp3) | -1.24 | | 0.0485 |
| Trib1 | tribbles pseudokinase 1 (Trib1) | -1.24 | | 0.0404 |
| F3 | coagulation factor III (F3) | -1.23 | | 0.0326 |
| Tgfbr1 | transforming growth factor, beta receptor I (Tgfbr1) | -1.23 | | 0.0297 |
| Fxr1 | FMR1 autosomal homolog 1 (Fxr1) | -1.23 | | 0.0395 |
| Mtss1 | MTSS I-BAR domain containing 1 (Mtss1) | -1.21 | | 0.0305 |
| Bmp2k | BMP2 inducible kinase (Bmp2k) | -1.21 | | 0.0368 |
| Pdha1 | pyruvate dehydrogenase E1 alpha 1 (Pdha1) | -1.21 | | 0.0193 |
| Map4k4 | mitogen-activated protein kinase kinase kinase kinase 4 (Map4k4) | -1.21 | | 0.0376 |
| Slc25a4 | solute carrier family 25 (mitochondrial carrier, adenine nucleotide translocator), member 4 (Slc25a4) | -1.20 | | 0.0444 |
| Tmed5 | transmembrane p24 trafficking protein 5 (Tmed5) | -1.19 | | 0.0362 |
| Jarid2 | jumonji, AT rich interactive domain 2 (Jarid2) | -1.19 | | 0.0343 |
| Igf1r | insulin-like growth factor I receptor (Igf1r) | -1.18 | | 0.0309 |
| Zfp36l1 | zinc finger protein 36, C3H type-like 1 (Zfp36l1) | -1.16 | | 0.0399 |
| Vps13b | vacuolar protein sorting 13B (Vps13b) | 1.18 | | 0.0474 |
| Fam83g | family with sequence similarity 83, member G (Fam83g) | 1.19 | | 0.0243 |
| Gm6548 | predicted gene 6548 (Gm6548) | 1.20 | | 0.0362 |
| Acsbg1 | acyl-CoA synthetase bubblegum family member 1 (Acsbg1) | 1.21 | | 0.0233 |
| Zadh2 | zinc binding alcohol dehydrogenase, domain containing 2 (Zadh2) | 1.21 | | 0.0476 |
| Ckmt1 | creatine kinase, mitochondrial 1, ubiquitous (Ckmt1) | 1.22 | | 0.0391 |
| Fam25c | family with sequence similarity 25, member C (Fam25c) | 1.22 | | 0.0370 |
| Cox6c | cytochrome c oxidase subunit 6C (Cox6c) | 1.22 | | 0.0380 |
| Cxadr | coxsackie virus and adenovirus receptor (Cxadr) | 1.24 | | 0.0275 |
| Cds1 | CDP-diacylglycerol synthase 1 (Cds1) | 1.24 | | 0.0414 |
| Fbxo45 | F-box protein 45 (Fbxo45) | 1.24 | | 0.0234 |
| Atp9b | ATPase, class II, type 9B (Atp9b) | 1.24 | | 0.0491 |
| Cpt1a | carnitine palmitoyltransferase 1a, liver (Cpt1a) | 1.24 | | 0.0113 |
| Pou3f1 | POU domain, class 3, transcription factor 1 (Pou3f1) | 1.25 | | 0.0129 |
| Slc26a2 | solute carrier family 26 (sulfate transporter), member 2 (Slc26a2) | 1.25 | | 0.0387 |
| Per3 | period circadian clock 3 (Per3) | 1.25 | | 0.0429 |
| Gm2a | GM2 ganglioside activator protein (Gm2a) | 1.26 | | 0.0190 |
| Hr | lysine demethylase and nuclear receptor corepressor (Hr) | 1.26 | | 0.0350 |
| Acad9 | acyl-Coenzyme A dehydrogenase family, member 9 (Acad9) | 1.26 | | 0.0260 |
| Them5 | thioesterase superfamily member 5 (Them5) | 1.26 | | 0.0344 |
| Lancl1 | LanC (bacterial lantibiotic synthetase component C)-like 1 (Lancl1) | 1.26 | | 0.0465 |
| Gjb5 | gap junction protein, beta 5 (Gjb5) | 1.26 | | 0.0332 |
| Hal | histidine ammonia lyase (Hal) | 1.27 | | 0.0376 |
| Zfp120 | zinc finger protein 120 (Zfp120) | 1.27 | | 0.0427 |
| Col6a3 | collagen, type VI, alpha 3 (Col6a3) | 1.28 | | 0.0235 |
| Fam219b | family with sequence similarity 219, member B (Fam219b) | 1.28 | | 0.0249 |
| Dtx2 | deltex 2, E3 ubiquitin ligase (Dtx2) | 1.30 | | 0.0150 |
| Col6a2 | collagen, type VI, alpha 2 (Col6a2) | 1.30 | | 0.0295 |
| Ifngr2 | interferon gamma receptor 2 (Ifngr2) | 1.30 | | 0.0163 |
| Mfap3l | microfibrillar-associated protein 3-like (Mfap3l) | 1.30 | | 0.0389 |
| Faah | fatty acid amide hydrolase (Faah) | 1.31 | | 0.0249 |
| Ercc4 | excision repair cross-complementing rodent repair deficiency, complementation group 4 (Ercc4) | 1.31 | | 0.0459 |
| Mrpl18 | mitochondrial ribosomal protein L18 (Mrpl18) | 1.31 | | 0.0491 |
| Zbtb7c | zinc finger and BTB domain containing 7C (Zbtb7c) | 1.32 | | 0.0376 |
| Psat1 | phosphoserine aminotransferase 1 (Psat1) | 1.33 | | 0.0176 |
| Snx11 | sorting nexin 11 (Snx11) | 1.33 | | 0.0285 |
| Trabd2b | TraB domain containing 2B (Trabd2b) | 1.33 | | 0.0108 |
| Aopep | aminopeptidase O (Aopep) | 1.34 | | 0.0211 |
| Oas1f | 2'-5' oligoadenylate synthetase 1F (Oas1f) | 1.34 | | 0.0425 |
| Ereg | epiregulin (Ereg) | 1.34 | | 0.0223 |
| Sgsm2 | small G protein signaling modulator 2 (Sgsm2) | 1.34 | | 0.0206 |
| Ing5 | inhibitor of growth family, member 5 (Ing5) | 1.35 | | 0.0239 |
| Fkbp10 | FK506 binding protein 10 (Fkbp10) | 1.35 | | 0.0242 |
| Ccdc88c | coiled-coil domain containing 88C (Ccdc88c) | 1.35 | | 0.0039 |
| Fam83f | family with sequence similarity 83, member F (Fam83f) | 1.36 | | 0.0047 |
| Col12a1 | collagen, type XII, alpha 1 (Col12a1) | 1.36 | | 0.0323 |
| Lce1e | late cornified envelope 1E (Lce1e) | 1.37 | | 0.0274 |
| Rnaseh2a | ribonuclease H2, large subunit (Rnaseh2a) | 1.37 | | 0.0179 |
| Wdr91 | WD repeat domain 91 (Wdr91) | 1.37 | | 0.0319 |
| Ywhaz | tyrosine 3-monooxygenase/tryptophan 5-monooxygenase activation protein, zeta polypeptide (Ywhaz) | 1.38 | | 0.0045 |
| Vrk2 | vaccinia related kinase 2 (Vrk2) | 1.38 | | 0.0482 |
| Neu3 | neuraminidase 3 (Neu3) | 1.38 | | 0.0451 |
| Lynx1 | Ly6/neurotoxin 1 (Lynx1) | 1.38 | | 0.0299 |
| Pla2g4f | phospholipase A2, group IVF (Pla2g4f) | 1.38 | | 0.0206 |
| Dgkq | diacylglycerol kinase, theta (Dgkq) | 1.39 | | 0.0363 |
| Mroh6 | maestro heat-like repeat family member 6 (Mroh6) | 1.40 | | 0.0326 |
| Slc2a5 | solute carrier family 2 (facilitated glucose transporter), member 5 (Slc2a5) | 1.40 | | 0.0370 |
| Atp6v1c2 | ATPase, H+ transporting, lysosomal V1 subunit C2 (Atp6v1c2) | 1.41 | | 0.0091 |
| 5031425E22Rik | RIKEN cDNA 4931406C07 gene (4931406C07Rik) | 1.41 | | 0.0213 |
| Atp12a | ATPase, H+/K+ transporting, nongastric, alpha polypeptide (Atp12a) | 1.41 | | 0.0367 |
| Sdr16c5 | short chain dehydrogenase/reductase family 16C, member 5 (Sdr16c5) | 1.42 | | 0.0351 |
| Col1a1 | collagen, type I, alpha 1 (Col1a1) | 1.43 | | 0.0360 |
| Rpl30 | ribosomal protein L30 (Rpl30) | 1.43 | | 0.0274 |
| E2f1 | E2F transcription factor 1 (E2f1) | 1.43 | | 0.0422 |
| Nkiras1 | NFKB inhibitor interacting Ras-like protein 1 (Nkiras1) | 1.43 | | 0.0345 |
| Asprv1 | aspartic peptidase, retroviral-like 1 (Asprv1) | 1.43 | | 0.0246 |
| Abhd12b | abhydrolase domain containing 12B (Abhd12b) | 1.44 | | 0.0195 |
| Pclaf | PCNA clamp associated factor (Pclaf) | 1.44 | | 0.0430 |
| Sfrp1 | secreted frizzled-related protein 1 (Sfrp1) | 1.45 | | 0.0052 |
| Sirt6 | sirtuin 6 (Sirt6) | 1.47 | | 0.0486 |
| Tspan15 | tetraspanin 15 (Tspan15) | 1.47 | | 0.0465 |
| Lrrc8b | leucine rich repeat containing 8 family, member B (Lrrc8b) | 1.47 | | 0.0017 |
| Lce1f | late cornified envelope 1F (Lce1f) | 1.47 | | 0.0321 |
| Sema4c | sema domain, immunoglobulin domain (Ig), transmembrane domain (TM) and short cytoplasmic domain, (semaphorin) 4C (Sema4c) | 1.51 | | 0.0037 |
| Chaf1b | chromatin assembly factor 1, subunit B (p60) (Chaf1b) | 1.51 | | 0.0491 |
| 9930012K11Rik | RIKEN cDNA 9930012K11 gene (9930012K11Rik) | 1.52 | | 0.0340 |
| Saysd1 | SAYSVFN motif domain containing 1 (Saysd1) | 1.53 | | 0.0494 |
| Slurp2 | secreted Ly6/Plaur domain containing 2 (Slurp2) | 1.53 | | 0.0379 |
| Nsg1 | neuron specific gene family member 1 (Nsg1) | 1.54 | | 0.0099 |
| Adh6a | alcohol dehydrogenase 6A (class V) (Adh6a) | 1.54 | | 0.0180 |
| Sox7 | SRY (sex determining region Y)-box 7 (Sox7) | 1.54 | | 0.0069 |
| Paqr3 | progestin and adipoQ receptor family member III (Paqr3) | 1.55 | | 0.0038 |
| Wwox | WW domain-containing oxidoreductase (Wwox) | 1.55 | | 0.0277 |
| Ankrd16 | ankyrin repeat domain 16 (Ankrd16) | 1.56 | | 0.0304 |
| Ggct | gamma-glutamyl cyclotransferase (Ggct) | 1.56 | | 0.0066 |
| Nipsnap1 | nipsnap homolog 1 (Nipsnap1) | 1.56 | | 0.0210 |
| Slurp1 | secreted Ly6/Plaur domain containing 1 (Slurp1) | 1.57 | | 0.0211 |
| Cntrob | centrobin, centrosomal BRCA2 interacting protein (Cntrob) | 1.57 | | 0.0431 |
| Tmem158 | transmembrane protein 158 (Tmem158) | 1.57 | | 0.0442 |
| Dlgap5 | DLG associated protein 5 (Dlgap5) | 1.58 | | 0.0267 |
| Tfap2e | transcription factor AP-2, epsilon (Tfap2e) | 1.58 | | 0.0174 |
| Calm4 | calmodulin 4 (Calm4) | 1.60 | | 0.0291 |
| Rab29 | RAB29, member RAS oncogene family (Rab29) | 1.61 | | 0.0388 |
| 9330162012Rik | RIKEN cDNA 5031425E22 gene (5031425E22Rik) | 1.61 | | 0.0229 |
| Dnase1l2 | deoxyribonuclease 1-like 2 (Dnase1l2) | 1.62 | | 0.0386 |
| Col27a1 | collagen, type XXVII, alpha 1 (Col27a1) | 1.63 | | 0.0085 |
| Slc6a20a | solute carrier family 6 (neurotransmitter transporter), member 20A (Slc6a20a) | 1.64 | | 0.0309 |
| Nemp2 | nuclear envelope integral membrane protein 2 (Nemp2) | 1.65 | | 0.0218 |
| Ndnf | neuron-derived neurotrophic factor (Ndnf) | 1.65 | | 0.0265 |
| Gmpr2 | guanosine monophosphate reductase 2 (Gmpr2) | 1.66 | | 0.0444 |
| Prrx2 | paired related homeobox 2 (Prrx2) | 1.66 | | 0.0435 |
| Gm16907 | predicted gene, 16907 (Gm16907) | 1.68 | | 0.0150 |
| Zfp825 | zinc finger protein 825 (Zfp825) | 1.68 | | 0.0189 |
| Lce6a | late cornified envelope 6A (Lce6a) | 1.69 | | 0.0040 |
| Fcgbp | Fc fragment of IgG binding protein (Fcgbp) | 1.69 | | 0.0008 |
| Pim2 | proviral integration site 2 (Pim2) | 1.70 | | 0.0288 |
| C1qtnf6 | C1q and tumor necrosis factor related protein 6 (C1qtnf6) | 1.70 | | 0.0228 |
| Slc34a2 | solute carrier family 34 (sodium phosphate), member 2 (Slc34a2) | 1.71 | | 0.0121 |
| Lce1g | late cornified envelope 1G (Lce1g) | 1.74 | | 0.0117 |
| Trim71 | tripartite motif-containing 71 (Trim71) | 1.77 | | 0.0357 |
| Stc2 | stanniocalcin 2 (Stc2) | 1.77 | | 0.0247 |
| Mrnip | MRN complex interacting protein (Mrnip) | 1.83 | | 0.0286 |
| Dio2 | deiodinase, iodothyronine, type II (Dio2) | 1.83 | | 0.0453 |
| Ccr9 | chemokine (C-C motif) receptor 9 (Ccr9) | 1.84 | | 0.0497 |
| Slc26a9 | solute carrier family 26, member 9 (Slc26a9) | 1.84 | | 0.0087 |
| Plod2 | procollagen lysine, 2-oxoglutarate 5-dioxygenase 2 (Plod2) | 1.85 | | 0.0271 |
| Rapgef4 | Rap guanine nucleotide exchange factor (GEF) 4 (Rapgef4) | 1.92 | | 0.0006 |
| Col22a1 | collagen, type XXII, alpha 1 (Col22a1) | 1.95 | | 0.0469 |
| Dsg1c | desmoglein 1 gamma (Dsg1c) | 1.97 | | 0.0033 |
| Xrcc3 | X-ray repair complementing defective repair in Chinese hamster cells 3 (Xrcc3) | 1.97 | | 0.0169 |
| 4930412O13Rik | RIKEN cDNA 1700037C18 gene (1700037C18Rik) | 1.99 | | 0.0322 |
| Tex15 | testis expressed gene 15 (Tex15) | 2.04 | | 0.0423 |
| Zfp385b | zinc finger protein 385B (Zfp385b) | 2.05 | | 0.0328 |
| Mnd1 | meiotic nuclear divisions 1 (Mnd1) | 2.06 | | 0.0201 |
| Postn | periostin, osteoblast specific factor (Postn) | 2.08 | | 0.0002 |
| Tle6 | transducin-like enhancer of split 6 (Tle6) | 2.08 | | 0.0414 |
| Snhg18 | small nucleolar RNA host gene 18 (Snhg18) | 2.09 | | 0.0284 |
| Jpx | Jpx transcript, Xist activator (non-protein coding) (Jpx) | 2.12 | | 0.0288 |
| Atp23 | ATP23 metallopeptidase and ATP synthase assembly factor homolog (Atp23) | 2.17 | | 0.0157 |
| Glipr1 | GLI pathogenesis-related 1 (glioma) (Glipr1) | 2.26 | | 0.0390 |
| A430088P11Rik | RIKEN cDNA A430088P11 gene (A430088P11Rik) | 2.27 | | 0.0245 |
| Tmem267 | transmembrane protein 45b (Tmem45b) | 2.29 | | 0.0027 |
| Col11a2 | collagen, type XI, alpha 2 (Col11a2) | 2.31 | | 0.0041 |
| Acat3 | acetyl-Coenzyme A acetyltransferase 3 (Acat3) | 2.39 | | 0.0281 |
| Bco2 | beta-carotene oxygenase 2 (Bco2) | 2.49 | | 0.0465 |
| Ccl22 | chemokine (C-C motif) ligand 22 (Ccl22) | 2.52 | | 0.0083 |
| Pyy | peptide YY (Pyy) | 2.58 | | 0.0270 |
| Calcb | calcitonin-related polypeptide, beta (Calcb) | 2.70 | | 0.0264 |
| Zmat4 | zinc finger, matrin type 4 (Zmat4) | 2.78 | | 0.0351 |
| Pcdhb4 | protocadherin beta 4 (Pcdhb4) | 2.81 | | 0.0234 |
| Gm45935 | predicted gene, 45935 (Gm45935) | 2.89 | | 0.0283 |
| Gpr21 | G protein-coupled receptor 21 (Gpr21) | 3.03 | | 0.0071 |
| Tmem200a | transmembrane protein 200A (Tmem200a) | 3.11 | | 0.0423 |
| Gsdmc2 | gasdermin C2 (Gsdmc2) | 3.16 | | 0.0162 |
| 1110028F11Rik | cDNA RIKEN 9330162012 gene (9330162012Rik) | 3.22 | | 0.0351 |
| Gm12191 | predicted gene 12191 (Gm12191) | 3.32 | | 0.0000 |
| Gm14391 | predicted gene 14391 (Gm14391) | 3.73 | | 0.0270 |
| Sprr2a3 | small proline-rich protein 2A3 (Sprr2a3) | 3.94 | | 0.0094 |
| Ceacam12 | CEA cell adhesion molecule 12 (Ceacam12) | 3.97 | | 0.0050 |
| Sprr2a1 | small proline-rich protein 2A1 (Sprr2a1) | 4.31 | | 0.0040 |
| Sprr2a2 | small proline-rich protein 2A2 (Sprr2a2) | 4.31 | | 0.0040 |
| Lpo | lactoperoxidase (Lpo) | 4.34 | | 0.0363 |

“-” indicates downregulation of genes in KO mice compared to that in WT mice.
