# Supplementary material for: Adiponectin in the mammalian host influences ticks’ acquisition of the Lyme disease pathogen Borrelia
Source: PLoS Biol. 2023 Oct 20;21(10):e3002331. doi: 10.1371/journal.pbio.3002331 (PMC10619873; doi:10.1371/journal.pbio.3002331)
Supplement: S3 Table — (DOCX) [file pbio.3002331.s007.docx]

**Table S3.** The primers used in this study.

| **Gene name** | **Primer sequence** |  |
| --- | --- | --- |
| Tick *actin* | F: GGCGACGTAGCAG R: GGTATCGTGCTCGACTC |  |
| Mouse *β-actin* | F: AGCGGGAAATCGTGCGTG  R: CAGGGTACATGGTGGTGCC |  |
| *Borrelia* *flaB* | F: TTCAATCAGGTAACGGCACA  R: GACGCRRGAGACCCTGAAAG |  |
| Adiponectin WT | F: TGGATGCTGCCATGTTCCCAT R: CTTGTGTCTGTGTCTAGGCCTT |  |
| Adiponectin KO | F: GGATGCTGCCATGTTCCCAT  R: CTCCAGACTGCCTTGGGA |  |
| HBP qPCR | F: GCGAGGAAAACGAGGAAAG  R: CTGAGGAGAAATGCGTATCG |  |
| TNF-α qPCR | F: AGGCACTCCCCCAAAAGATG R: TGGTGGTTTGTGAGTGTGAGG |  |
|  |  |  |
| IL-18 qPCR | F: GACTCTTGCGTCAACTTCAAGG R: CAGGCTGTCTTTTGTCAACGA |  |
|  |  |  |
| IL-6 qPCR | F: ATACCACTCCCAACAGACCT R: CCAGTTTGGTAGCATCCATC |  |
|  |  |  |
| IL-1β qPCR | F: GCAGTGGTTCGAGGCCTAAT R: GCTGCTTCAGACACTTGCAC |  |
|  |  |  |
| CCL3 qPCR | F: GCCAGGTGTCATTTTCCTGAC R: CTCAAGCCCCTGCTCTACAC |  |
|  |  |  |
| IFN-γ qPCR | F: GAGGAACTGGCAAAAGGATGG R: ACCTGTGGGTTGTTGACCTC |  |
|  |  |  |
| CCL5 qPCR | F: GACAGCACATGCATCTCCCA R: GTGTCCGAGCCATATGGTGA |  |
|  |  |  |
| IL-10 qPCR | F: GTACAGCCGGGAAGACAATAAC R: GCATTAAGGAGTCGGTTAGCAG |  |
|  |  |  |
| TLR2 qPCR | F: AAGAGGAAGCCCAAGAAAGC R: AATGGGAATCCTGCTCACTG |  |
|  |  |  |
| IL-4 qPCR | F: CGGATGCGACAAAAATCAC R: CGTTTGGCACATCCATCTC |  |
|  |  |  |
| IL-12 qPCR | F: ATCGTTTTGCTGGTGTCTCC R: CTTCTTCAGGCGTGTCACAG |  |
|  |  |  |
| TGF-β qPCR | F: TGGAGCAACATGTGGAACTC R: TGCCGTACAACTCCAGTGAC |  |
|  |  |  |
| IL-17 qPCR | F: TCATCTGTGTCTCTGATGCTGTTG R: TCGCTGCCTTCACTGT |  |
|  |  |  |
