# Supplementary material for: Adiponectin in the mammalian host influences ticks’ acquisition of the Lyme disease pathogen Borrelia
Source: PLoS Biol. 2023 Oct 20;21(10):e3002331. doi: 10.1371/journal.pbio.3002331 (PMC10619873; doi:10.1371/journal.pbio.3002331)
Supplement: Attachment — Submitted filename: Response to Reviewers.docx [file pbio.3002331.s010.docx]

**Response to academic editor**

Thank you for your patience while your manuscript "Adiponectin inhibits acquisition of the Lyme disease agent by ticks" was peer-reviewed at PLOS Biology. It has now been evaluated by the PLOS Biology editors, an Academic Editor with relevant expertise, and by several independent reviewers. In light of the reviews, which you will find at the end of this email along with the comments from the Academic Editor, we would like to invite you to revise the work to thoroughly address the reviewers' reports.

We would like to thank the editor and all the reviewers for providing thoughtful insights to improve our manuscript. We also greatly appreciate your interest in our study. Please see below for the point-to-point response.

As you will see below, the reviewers have important issues that should be solved before further consideration. In particular, the reviewers think that some of your conclusions are overstatements, the manuscript is in need of clarifications and in need of adding information to the methods. Regarding this last point, it is very important that you add the number of mice, bacteria and ticks, in particular the bacterial burden in the in vivo models. Reviewer #1 finds the histopathology data inadequate. All those issues will need to be solved before further consideration of the manuscript.

We agree with all the comments. (1) We have edited the statement and conclusion appropriately according to the reviewers’ kind suggestions. To be more accurate, we also changed our title to “Adiponectin deficiency alters acquisition of the Lyme disease agent by ticks”. (2) We have clarified and added more details associated with methods, including the numbers of mice, bacteria, and ticks. We also added the numbers throughout the manuscript, including methods, results, and all the figure legends. (3) We have added the bacterial burden in mice as new Fig. 1A. (4) We have also provided a histopathology image in Fig. 3 and added the histopathology scoring system details in the methods. All the suggestions provided by the editor and reviewers greatly improve our manuscript. Thank you.

COMMENTS FROM THE ACADEMIC EDITOR:

I cannot judge the need for additional experiments regarding histopathology because this is outside my area of technical expertise. However, I do agree with several key points raised by Reviewer 1, which overlap with some of the comments made by the other two reviewers.

- First, there is a general issue of overinterpretation. The conclusion that adiponectin influences Borrelia acquisition by ticks is overstated in that the direct effect of adiponectin is not truly measured. The difference between WT and adiponectin-deficient mice could be due to other factors associated with adiponectin deficiency rather than directly due to differences in adiponectin levels in the blood meal. Also, the claim that "adiponectin influences B. burgdorferi acquisition by ticks, which is the first evidence that a host factor in blood modulates pathogen acquisition by arthropod vector" (lines 219-221) is untrue. The authors themselves explain in the introduction that host blood metabolites can directly or indirectly influence arthropod susceptibility to pathogens. They cite the example of the host-derived cytokine in blood IFN-γ, which interacts with tick receptor Dome1 and activates the STAT-dependent pathway, limiting B. burgdorferi persistence in Ixodes scapularis.

Thank you for your comments. First, we have provided an image of the histopathology in Fig. 3 and added the histopathology scoring system details in the methods.

Second, we have edited the statement and conclusion appropriately and added wording regarding “adiponectin associated factors”. To be more accurate, we also changed our title to “Adiponectin deficiency alters acquisition of the Lyme disease agent by ticks”.

Third, we appreciate the comments on host factors in blood modulates pathogen acquisition by arthropod vector. In general, the studies we cited are all the findings that host factors influence pathogen colonization, which is a process that mainly happens at the vector midgut by affecting immune and/or physiological status of the vector. In our study, we find that host factor modulates pathogen acquisition by vector, which mostly happen at the interface of vector and host, and in our case is the tick bite site. Therefore, colonization and acquisition are two distinct processes. However, we fully agree that this can be confusing and that it is better to be more cautious in our description. Therefore, we have modified the text and deleted the claim, according to your suggestion.

- Second, a critical piece of information is missing. The authors state that WT and adiponectin-deficient mice had "a similar B. burgdorferi burden" (line 102) but they did not show the data supporting this statement. This information is essential and must be duly justified. It should include a measure of Borrelia survival at the tick bite site.

Thank you for this comment. We agree that this is very important. We have now provided the *B. burgdorferi* burden in WT and KO mice as Fig. 1A. Adiponectin also had no direct effect on *B. burgdorferi* viability as we show in Fig. 2B. As differences in immune cells and the level of inflammation (Fig. 3) at the tick bite site were observed in the WT and KO mice, we also measured *B. burgdorferi* survival at the tick bite site through culturing the skin biopsies of tick bite site and assessment using the BacTiter-Glo microbial cell viability assay. After 25 days culturing, we found *B. burgdorferi* in the tick-bite biopsy from KO mice has higher total viability than in WT mice in the positive samples. These data are shown in Fig. S4A. Thank you so much for summarizing and providing key comments to improve our manuscript.

**Response to Reviewer #1**

The authors make the argument that adiponectin, an abundant adipocyte-derived hormone in humans, inhibits Ixodes scapularis acquisition of Borrelia burgdorferi, the agent of Lyme disease.  The authors first demonstrate that B. burgdorferi levels are increased in ticks fed on adiponectin deficient mice compared to wild type mice and attribute elevated mRNA for three tick genes to adiponectin.  The investigators then compare various aspects of the tick feeding site between C57BL/6 adiponectin deficient and C57BL/6 wild type mice. The data suggest that adiponectin induces host histamine release in response to tick feeding, which in turn leads to vascular leakage.  The adiponectin deficient mice also have reduced numbers of neutrophils, macrophages, and inflammation at the bite site as well as reduced IL-12 and IL-1b. The possible effect of adiponectin on tick acquisition of B. burgdorferi is a potentially important finding.
With a few exceptions, the conclusions are supported by the data. The authors do overstate causation when correlation would be more appropriate. Specific examples are listed below. The histopathology data is inadequate. The methods require some additional details and clarification.

We appreciate your thoughtful insights to improve our manuscript. We agree with all your comments and have carefully revised the manuscript accordingly. Please see a point-to-point response below.

Line 41 Abstract: The authors state that, "All these factors mediated by adiponectin influence B.burgdorferi survival at the interface of tick bite site. This study represents a conceptual advancement that a host adipocyte-derived hormone modulates pathogen acquisition by a blood feeding arthropod." These conclusions are somewhat overstated in that the direct effect of adiponectin is not truly measured nor is survival of the B. burgdorferi at the tick bite site.

We agree with the comment. This is a very important point. To be more accurate and careful, we have revised the statement according to your suggestion as below:

“Here we report that adiponectin, the most abundant adipocyte-derived hormone circulating in human blood, **directly or indirectly** inhibits acquisition of the Lyme disease agent, *Borrelia burgdorferi*, by *Ixodes scapularis* ticks.”

“All these factors mediated by adiponectin or **associated factors** influence *B. burgdorferi* survival at the tick bite site. These results suggest a host adipocyte-derived hormone modulates pathogen acquisition by a blood feeding arthropod.”

We have also provided new data by culturing spirochetes at the skin biopsy site and then quantified the *B. burgdorferi* burden. We have evidence showing that inflammation (Fig. 3) at the tick bite site may affect *B. burgdorferi* survival. These data are shown in Fig. S4A and in the text as stated below:

“Since differences in immune cells and the level of inflammation at the tick bite site were observed between the WT and KO mice, we further evaluated *B. burgdorferi* survival at the tick bite site by culturing the skin biopsy specimens from WT and KO mice (n = 4). In total, 19 and 18 tick-bite biopsy specimens were collected from WT and KO mice, respectively. After culturing for 25 days, 7 (out of 19) and 8 (out of 18) skin samples from tick attachment sites of WT and KO mice were *B. burgdorferi*-positive without contamination, respectively. Within the positive samples, the *B. burgdorferi* viability was further measured using a BacTiter-Glo microbial cell viability assay. Indeed, we found *B. burgdorferi* in the tick-bite biopsy from KO mice has higher total viability than in WT mice (*p* < 0.05) (Fig. S4A).”

Line 103: The authors state that B. burgdorferi levels were equivalent in the WT and adiponectin deficient mice.  The number of mice used in this experiment should be reported.  Additionally, the actual quantity of B. burgdorferi in the mice is essential for interpreting the difference in B. burgdorferi levels in tick midguts and thus should be reported.

Thank you for your comments. We have added the numbers of animals and ticks throughout the manuscript, including the methods, results, and all the figure legends. We have also provided the *Borrelia* burden in the mice as a new Fig. 1A.

Line 121: The authors state that, "We examined whether incoming blood adiponectin influences tick physiology or alters the local host environment at the tick bite site."  The authors do not demonstrate that there is measurable adiponectin in the blood meal of the wildtype mice.  Thus, the changes in tick gene expression may be due to other factors associated with adiponectin deficiency rather than directly due to differences in adiponectin levels. This conclusion needs to be more accurately stated.

We agree with the comment. We have changed the statement to “We examined whether incoming blood adiponectin **or associated factors** influence tick physiology or alters the local host environment at the tick bite site.” We have added the other factors associated with adiponectin deficiency throughout the manuscript. To be more accurate, we also changed the title to “Adiponectin deficiency alters acquisition of the Lyme disease agent by ticks”.

Line 238: The authors state, "In our study, higher levels of histamine in WT mice increased vascular leakage, which led to the additional recruitment of neutrophils and macrophages to the tick bite site and caused severe inflammation." This is an over statement of causation. There are many other factors besides vascular permeability that can lead to recruitment of neutrophils and macrophages. This conclusion should be more accurately stated and the other possible causes of increased neutrophils and macrophage at the bite site should be addressed.

We agree. We have changed the statement to “In our study, increased vascular leakage was observed in WT mice. The vascular leakage and/or **other associated factors** may lead to the additional recruitment of neutrophils and macrophages to the tick bite site, resulting in severe inflammation.”

Line 254. The authors state, "In summary, we have discovered that host serum adiponectin affects the initial entry of B. burgdorferi into ticks."  This is an overinterpretation of the data. Please edit appropriately.

Thank you for the suggestion. We have edited the statement so that it states, “In summary, we have discovered that host serum adiponectin **or its associated factors** may affect the initial entry of *B. burgdorferi* into ticks.”

Methods and Results: Number of mice used in each experiment should be stated.
Line 290: Please clarify how qPCR analysis was done to enumerate B. burgdorferi levels in the tick midgut. Presumably this was done using genomic DNA, please clarify. It seems unusual to report ng of nucleic acid when the readout from qPCR is either copy number or relative expression (if using mRNA). Please describe how the data analysis was done to determine ng of amplicon was determined.

We have added the numbers of mice throughout the manuscript, including the methods, results, and all the figure legends.

We have also clarified the quantification method used in the manuscript.

“The primers for qPCR are listed in Table S3. The *B. burgdorferi* burden was estimated with a method described in our previous studies (references below). Specifically, *B. burgdorferi flagellin* gene (*flab*), mouse *actin* gene, and tick *actin* gene primers (Table S3) were first used to amplify the *flab* and *actin* gene, respectively. The concentration of PCR products was then quantified using NanoDrop 2000/2000c Spectrophotometers (Thermo Scientific, # ND2000CLAPTOP). The standard curve of qPCR Cq value and amplicon amount was derived from a series of known DNA dilutions of each target gene. The *B. burgdorferi* burden was quantified by extrapolation from the standard curve and the data were normalized to mouse or tick actin, and reported as ng *flab*/ 1000 ng *actin*.”

References:

Cao, Yongguo, et al. "An *Ixodes scapularis* protein disulfide isomerase contributes to *Borrelia burgdorferi* colonization of the vector." Infection and Immunity 88.12 (2020): 10-1128.

Narasimhan, Sukanya, et al. "Modulation of the tick gut milieu by a secreted tick protein favors *Borrelia burgdorferi* colonization." Nature Communications 8.1 (2017): 184.

Narasimhan, Sukanya, et al. "A tick gut protein with fibronectin III domains aids *Borrelia burgdorferi* congregation to the gut during transmission." PLoS Pathogens 10.8 (2014): e1004278.

Fig 1A - should indicate that the mice were needle inoculated with B. burgdorferi.

We have added this information in the results section and figure 1 legend.

Line 288: The oligonucleotides for PCR should be included.

We have provided the oligonucleotides/primers for PCR as Table S3. Thank you.

Line 311: The title for the section "Splenocyte viability evaluation" requires editing for accuracy or clarity.

Thank you for your careful review. We have corrected it.

Line 359: Was the tick bite site or the ears used for immune cell analysis?  Please clarify.

The tick bite site punch biopsy (not the whole ear) was used for immune cell analysis. We have clarified this in the text. Thank you.

Line 380: Please provide more details about the histopathology scoring system.  How were sections selected for evaluation? How many sections were evaluated?  Please clarify the criteria for minimal, mild, moderate, marked and severe?  Presumably this is inflammation, but this should be stated. Please include representative images or remove these data.

Thank you for these important suggestions. We have provided images of histopathology in Fig. 3 and more details about score system in the manuscript.

“Five WT and 4 KO mice with attached ticks were euthanized as described above 96 hours post tick placement and the ears (n = 18) were removed at the base of the ear and placed in 10% neutral buffered formalin and submitted to the Comparative Pathology Research (CPR) Core, Histology Laboratory (Department of Comparative Medicine, Yale University School of Medicine) for routine processing into paraffin wax where the ears were subsequently “bread-loafed” into 3-5 strips prior to embedding as previously described. The tissue blocks were sectioned at 5 microns and stained for hematoxylin and eosin (HE) by routine methods. The 18 HE-stained slides were evaluated blindly to the experimental manipulation (CJB) and semiquantitative analysis as described in Kurokawa et al, 2020. Specifically, each ear was evaluated for the presence or absence of ticks, the number of bite/inflammatory foci per slide. Each bite/inflammatory foci were scored for edema, hemorrhage, fibrin/necrosis, and inflammation where 5 = severe, 4 = marked, 3 = moderate, 2 = mild, 1 = minimal, scant 0.5, and trace = 0.25. The individual parameters were averaged separately as well as the sum all parameters for each tick bite to give a severity of injury score for each tick bite. The slides were evaluated using an Olympus BX 53 light microscope, photomicrographs taken using an Olympus DP28 camera using Olympus cellSens 4.1 imaging software (Olympus, Evident Scientific, Dallas, TX), and the images optimized using Adobe Photoshop Creative Cloud 23.0.0 (Adobe Inc., San Jose, CA).”

Reference: Kurokawa, Cheyne, et al. "Repeat tick exposure elicits distinct immune responses in guinea pigs and mice." Ticks and tick-borne diseases 11.6 (2020): 101529.

**Response to Reviewer #2**

The manuscript "Adiponectin inhibits acquisition of Lyme disease agent by ticks" was a pleasure to read and presents potentially impactful findings. Further, many findings of this paper open more questions ripe for future investigation. As interesting as I find the study, I would like a few points addressed.

We appreciate your interest in our study and thank you for the careful review to improve our manuscript. Please see the point-to-point response below.

The Authors report that ticks fed on infected adipo -/- mice had significantly elevated Borrelia acquisition compared to feeding on WT mice. However, this did not impact the feeding success of ticks. One key detail that is stated, but not shown (Line 101-102 Figure A), is the level of Borrelia in the mice. Figure A shows a diagram of the experiment, but the bacterial burden in the mice is missing. The bacterial burden from these mice groups should be included to show the acquisition difference is not reflecting the burden in the mice.

Thank you for your comment. This is very important. We have provided the *Borrelia* burden in the mice as a new Fig. 1A.

Numbers of animals need to be stated throughout for the reader to judge rigor of each experiment.

Thank you very much for this suggestion. We have added the numbers of animals and ticks throughout the manuscript, including the methods, results, and all the figure legends.

Please include the numbers of ticks and mice used in Fig1B,C,D.  Are these all the same mice, or are different mice in A,B,C. If they are stating the engorgement weight does not differ, I would like to see weights of the ticks used in FigB. Just from counting dots on the graph, it appears there are more ticks in Fig 1D.

Thank you for your comment. We have provided the numbers of ticks and mice according to your suggestion. The original Fig. 1B is an independent experiment. We didn’t weight the tick as they are 48 h post feeding. The original Figs. 1C and 1D are from the same experiment.

Are the mice used in Fig1 C, D infected?

The mice from original Figs. 1 C and 1D were infected. We have added the information.

From the Fig1B graph it appears that only 12 ticks are in the adipo-/- group. How many mice are these are these ticks from, how many ticks per mouse fed?

In this experiment, three infected mice with similar *B. burgdorferi* burden were fed upon by ticks. We put 10 ticks on each mouse at the beginning. In this experiment, some ticks dropped off or escaped from mice as the experiment progressed, and this is expected. We could collect an average of 4 ticks from each mouse for analysis at the end. We have provided the information in the method and figure legend.

To investigate what may be causing the increased Borrelia acquisition in the adiponectin -/- fed ticks they performed RNAseq on ticks collected from wt. and adipose -/- mice. From this data, they found histamine binding proteins were differentially expressed between wt. and adiponectin -/- fed ticks. They found that adiponectin -/- fed ticks had lower levels transcription of HBPs. They did not explore the downstream impacts of these HBPs in the tick, but instead it prompted them to investigate the levels of histamine between WT and adipo-/- mice. A strength, but also a weakness, in their RNA seq data is that it was done in the absence of Borrelia infection. This does isolate the variable of adipo-/- better but also lessens the direct relevance to Borrelia acquisition. I would have liked to see an experiment showing the HBP expression differences hold in ticks fed on infected adipo-/- vs wt. mice. If not part of RNAseq study, could transcription of the HBP genes be measured by qPCR from the tick RNA samples used in figure 1B,1D?
The differences in HBP expression prompted them to investigate the levels of histamine, and its downstream consequences at the tick bite site. They showed that the adipo-/- mice secreted less histamine, resulting in less immune cell infiltration and attenuated inflammation at the bite site. These results demonstrate that the adipo-/- mice here a greatly dampened immune reaction at the bite site, which is consistent with the increased Borrelia acquisition by ticks from the adipo-/- ticks.

Thank you for this very important suggestion. We have evaluated the HBP expression in the tick samples from original Fig. 1B according to your suggestion. We did not test the samples from the original Fig. 1D as we didn’t extract their RNA and the ticks had already molted to adults. From the samples in the original Fig. 1B, we still can find HBP expression differences in the ticks the fed on the infected WT and KO mice. These data are shown below. We have also added a histopathology image showing difference of inflammation at the tick bite site between WT and KO mice in Fig. 3 for your reference.

These findings are consistent with the higher Borrelia acquisition by ticks fed on adipo-/- mice, however they have not directly tested the impact of adiponectin dependent histamine levels on Borrelia acquisition. Could a rescue experiment address this? Could infected adipo-/- mice be supplemented to stimulate histamine release, and/or could WT mice be treated with antihistamines, to see if it is reduces the differences in Borrelia acquisition. Alternatively, if the effect of histamine on Borrelia acquisition was directly tested previously, an expanded discussion could address this concern.

Thank you for your kind suggestion. The rescue experiment is a good idea. We intradermally injected 0.3 ug/mL anti-dinitrophenyl (DNP) IgE in KO mice according to Ashina et al. (2015) to stimulate histamine release. We still found higher *B. burgdorferi* burden in ticks feeding on KO mice, but the difference is not significant (*p* = 0.0767). We have included this data as Fig. S4B in the manuscript. Thank you so much for providing all the valuable comments to improve our manuscript.

Reference:

Ashina, Kohei, et al. "Histamine induces vascular hyperpermeability by increasing blood flow and endothelial barrier disruption in vivo." Plos one 10.7 (2015): e0132367.

**Response to Reviewer #3**

I have only a few suggested revisions/questions regarding this innovative and well-designed study.

Thank you so much for your interest in our study. We greatly appreciate your careful review to improve our manuscript. Please see below for the point-to-point response.

1. Line 72-74. Why would a high concentration of adiponectin per se cause varied functions in multiple organs? Might moderate or even low concentrations cause varied functions in multiple organs?

We agree. The adiponectin concentration in blood is relatively high. To be cautious, we have changed the sentence as follows: Adiponectin have varied functions in multiple organs, including the liver, heart, and kidney.

2. Line 102. It would be useful to mention here how experimental and control mice were infected that ensured a similar B. burgdorferi burden even though the infection protocol is described in the Methods section of the paper.

Thank you for this suggestion. We have added the information that WT and KO animals were first needle inoculated with *B. burgdorferi*. The infected mice with similar *B. burgdorferi burden* were then subjected to being fed upon by ticks.

3. Line 111-112. How does the hormone adiponectin encode a C1q domain?

The full-length adiponectin contains an NH2-terminal hyper-variable region, a collagenous domain, and COOH-terminal C1q-like globular domain. We added the C1q-like globular domain in the manuscript.

4. Lines 248-249. Are adiponectin levels affected by the amount of B. burgdorferi introduced by tick bite and/or the number tick bite exposures in the mammalian host?

Thank you for your comments. We have data showing that needle inoculated *B. burgdorferi* infection has no effect on adiponectin levels in mice. It would also be interesting to test whether adiponectin levels are affected by tick-transmitted *B. burgdorferi* and/or the number of tick bite exposures in the future.

5. Line 274-276. How does the injection of 1 x 105 cells/ml B. burgdorferi compare with the amount of B. burgdorferi introduced during I. scapularis nymphal feeding and does it matter in regard to experimental outcomes?

This is a good point. The injection of 1 x 10^5^ cells/ml *B. burgdorferi* is enough to infect mice. Importantly, it is a standard amount to infect mice and has been adopted by many studies. The exact amount of *B. burgdorferi* introduced into mice by nymphal ticks is more variable and estimates differ among researchers, since there are variations among individual ticks. Most individuals suggest that between 10^2^ and 10^4^ spirochetes are transmitted following a tick bite. This is not likely to affect our experimental outcomes since our adiponectin WT and KO mice were infected by *B. burgdorferi* together with the same treatment by needle-injection.

6. Lines 379-381. Does this sentence describe the inflammation severity score? If so, was a more objective quantitative unstated definition used to assess the score (e.g., number inflammatory cells for each severity category)?

Thank you so much for your comments. We used a semiquantitative analysis. We provide the histopathology images in Fig. 3 and more details about score system in the method section.

“Specifically, each sample was evaluated for the presence or absence of ticks, and the number of bite/inflammatory foci per slide. Each bite/inflammatory foci was scored for edema, hemorrhage, fibrin/necrosis, and inflammation where 5 = severe, 4 = marked, 3 = moderate, 2 = mild, 1 = minimal, scant 0.5, and trace = 0.25. The individual parameters were averaged separately as well as the sum all parameters for each tick bite to give a severity of injury score for each tick bite.”

7. Figure 3B. The pink arrows mentioned in the Figure legend are difficult to visualize in Figure 3B.

Thanks for pointing it out. Dr. Carmen, one of the authors, changed the arrows to circle.

Minor
8. Line 114. Substitute "directly" for "direct".

Done accordingly.

9. Line 143. Substitute "levels" for "level".

Done accordingly.

10. Line 219. Suggest revising the sentence to read: "In this study, we found that host derived adiponectin influences B. burgdorferi acquisition by ticks, which is the first evidence that a host factor in blood modulates pathogen acquisition by arthropod vector.

Done accordingly.

11. Line 258. Substitute " modulate" for "modulates"

Done accordingly.

12. Line 396.  Substitute "Reactive oxygen species (ROS) quantification…" for "ROS quantification…"

Done accordingly.

13. Line 611. Substitute "…at the tick bite site of WT…" for "at the tick bite of WT…"

Done accordingly.

14. Line 572 (Figure 1A legend). Substitute "(A) Pathogen-free I. scapularis nymphs
were fed on…    for "(A) Ticks were fed on…"

Done accordingly. Thank you so much for your careful review and comments to improve our manuscript.
